# Effects of chameleon dispense-to-plunge speed on particle concentration, complex formation, and final resolution: A case study using the *Neisseria gonorrhoeae* ribonucleotide reductase inactive complex

**DOI:** 10.1016/j.jsb.2021.107825

**Published:** 2021-12-11

**Authors:** Talya S. Levitz, Edward J. Brignole, Ivan Fong, Michele C. Darrow, Catherine L. Drennan

**Affiliations:** a Department of Biology, Massachusetts Institute of Technology, 77 Massachusetts Avenue, Cambridge, MA, USA; b MIT.nano, Massachusetts Institute of Technology, 77 Massachusetts Avenue, Cambridge, MA, USA; c SPT Labtech Melbourn Science Park, Cambridge Rd, Melbourn SG8 6HB, United Kingdom; d Department of Chemistry, Massachusetts Institute of Technology, 77 Massachusetts Avenue, Cambridge, MA, USA; e Howard Hughes Medical Institute, Massachusetts Institute of Technology, Cambridge, MA, USA

**Keywords:** Cryo-EM, Dispense-to-plunge time, chameleon, Ribonucleotide reductase, Air/water interface, Particle denaturation

## Abstract

Ribonucleotide reductase (RNR) is an essential enzyme that converts ribonucleotides to deoxyribonucleotides and is a promising antibiotic target, but few RNRs have been structurally characterized. We present the use of the chameleon, a commercially-available piezoelectric cryogenic electron microscopy plunger, to address complex denaturation in the *Neisseria gonorrhoeae* class Ia RNR. Here, we characterize the extent of denaturation of the ring-shaped complex following grid preparation using a traditional plunger and using a chameleon with varying dispense-to-plunge times. We also characterize how dispense-to-plunge time influences the amount of protein sample required for grid preparation and preferred orientation of the sample. We demonstrate that the fastest dispense-to-plunge time of 54 ms is sufficient for generation of a data set that produces a high quality structure, and that a traditional plunging technique or slow chameleon dispense-to-plunge times generate data sets limited in resolution by complex denaturation. The 4.3 Å resolution structure of *Neisseria gonorrhoeae* class Ia RNR in the inactive α4β4 oligomeric state solved using the chameleon with a fast dispense-to-plunge time yields molecular information regarding similarities and differences to the well studied *Escherichia coli* class Ia RNR α4β4 ring.

## 1. Introduction

Cryogenic electron microscopy (cryo-EM) has rapidly joined X-ray crystallography and NMR as a leading technique for structural determination of proteins. In the last two decades, development of direct electron detectors, improvements in automated sample loading and acquisition, and development of user-friendly software have led to an explosion of cryo-EM structures in the Protein Data Bank (reviewed in Cheng, 2018; Lyumkis, 2019; Nogales, 2016). However, sample preparation is still a cryo-EM bottleneck (Lyumkis, 2019); one common hurdle is proteins preferentially adsorbing to the air/water interface, which can manifest as particle denaturation and/or preferred orientation (Glaeser, 2018; Noble et al., 2018a,b). Using conventional blotting and plunging techniques, even without factoring in interface adsorption, each protein can contact the air/water interface thousands of times during the grid preparation process, and the filter paper itself may exacerbate denaturation effects (Armstrong et al., 2020; D’Imprima et al., 2019; Taylor and Glaeser, 2008).

In order to ameliorate some of the denaturation and preferred orientation effects, Carragher et al. developed the Spotiton, a cryo-EM grid preparation instrument that uses the piezoelectric effect to spray protein onto self-wicking grids (Dandey et al., 2018; Jain et al., 2012; Razinkov et al., 2016; Wei et al., 2018). The Spotiton can assist with preferred orientation and, due to a fast dispense-to-plunge time, reduces the number of contacts each particle makes with the air/water interface (Noble et al., 2018a,b). The commercially-available successor to the Spotiton, the chameleon (developed by SPT Labtech), allows for precise user control of dispense-to-plunge time, a parameter that can be tuned through glow discharge of commercially-available nanowire grids (Darrow et al., 2019; Wei et al., 2019). Additional devices with alternative methods of dispensing or wicking sample, including the Vitrojet (Ravelli et al., 2020), CryoWriter (Schmidli et al., 2019), Shake-it-off (Rubinstein et al., 2019), Back-it-up (Tan and Rubinstein, 2020), time-resolved sample preparation (TrEM) (Mäeots et al., 2020), and an automated cryo-plunger for CLEM and cryo-fluorescence from Linkam (Kamp et al., 2020) are also in development and show promise for expansion of the cryo-EM field.

Here, we use the chameleon to systematically study the effects of dispense-to-plunge time on complex denaturation and preferred orientation of the inactivated ribonucleotide reductase (RNR) complex from *Neisseria gonorrhoeae.* RNR is an essential enzyme found in almost every organism that converts ribonucleotides to deoxyribonucleotides. The first-identified and most-studied class of RNRs, the class Ia RNRs, uses a diiron-oxo center to generate a stable tyrosyl radical in the radical-generating β subunit that travels >30 Å to the active site in the catalytic α subunit to remove the 2′-hydroxyl group from the ribonucleotide diphosphate substrate (Kang et al., 2020; Reece and Seyedsayamdost, 2017; Seyedsayamdost et al., 2007). In order to respond to the deoxyribonucleotide needs of the organism, class Ia RNRs have two allosteric sites in addition to the catalytic site: the specificity site, which determines which of the four ribonucleotide substrates is bound in the active site; and the activity site, which acts as a general on/off switch for the enzyme (Brown and Reichard, 1969; Eriksson et al., 1997). When dATP is bound in the activity site, class Ia RNR enzymes have been shown to form ring-shaped structures that physically prevent radical transfer between the β and α subunits; when ATP displaces dATP, the β and α subunits can come together to form an active α2β2 complex (Ando et al., 2011; Kang et al., 2020). Although all class Ia RNRs studied to date form ring-shaped inactive structures in the presence of dATP, the compositions of the rings vary, with the human enzyme forming α6 inhibited rings whereas the *Escherichia coli* enzyme forms α4β4 inhibited rings (Ando et al., 2016, 2011; Brignole et al., 2018; Fairman et al., 2011; Zimanyi et al., 2012).

Unlike some bacteria that have multiple classes of RNR enzymes, *N. gonorrhoeae* only have one RNR, a class Ia enzyme. This RNR is essential for *N. gonorrhoeae*, making it a potential antibiotic target in an organism for which the need for novel antibiotics is pressing (CDC, 2018; Remmele et al., 2014). We recently demonstrated using negative stain electron microscopy that, similar to the homologous *E. coli* RNR, the *N. gonorrhoeae* RNR forms an α4β4 inactive complex (Narasimhan et al., 2020). The ring-shaped inactive state predisposes the complex to preferred orientation problems but also allows for easy detection of complex denaturation, making it a challenging cryo-EM sample but one for which screening for optimal conditions is rapid. Here, we show that the fastest chameleon dispense-to-plunge time of 54 ms generates a cryo-EM structure at a resolution unattainable using a traditional blotting-based plunging technique or slower dispense-to-plunge speeds.

## 2. Materials and methods

### 2.1. Protein cloning and purification

The *N. gonorrhoeae* RNR NrdA (α) and NrdB (β) genes with an N-terminal hexahistidine (His6) tag followed by a TEV cleavage site (ENLYFQ) were separately inserted into pET30a(+) vectors under control of a T7 promoter. Cloning was done using Gibson assembly (components from NEB) and the primers for backbone amplification were purchased from Sigma (Gibson et al., 2009). Primer sequences for amplification of the pET30a(+) backbone prior to Gibson assembly of each construct are listed in Supplementary Table 1 and gene block sequences inserted into the pET30a(+) backbone are listed in Supplementary Table 2. Codon optimization of all genes was completed using the IDT online calculator prior to purchase (Integrated DNA Technologies). The sequence of each insert was confirmed through DNA sequencing (Quintara Biosciences).

His_6_-TEV-*Ng*RNR α and His_6_-TEV-*Ng*RNR β were expressed from plasmid pET30a(+) in *E. coli* strain T7 Express (NEB). Expression began with a 1:100 inoculation of overnight culture grown from a single colony into 1 L LB + 50 ng/μL kanamycin. The cultures were grown at 37 °C with shaking at 220 rpm until OD_600_ reached 0.6–0.8, at which point the cells were moved to 18 °C before induction with 1 mM IPTG. Cells continued shaking overnight at 18 °C (approx. 20–22 h) before being spun down at 4000*g* for 15 min at 4 °C and frozen at −20 °C until lysis and purification. The proteins were purified based on previously described methods for purification of *E. coli* α2 with the modifications detailed below (Chen et al., 2018). Briefly, cells were resuspended in 30 mL resuspension buffer (50 mM NaH_2_PO_4_ pH 8.0, 500 mM NaCl, 10 mM imidazole, 10% [v/v] glycerol, and 5 mM β-mercaptoethanol), lysed by sonication, and clarified by centrifugation at 25,000 xg. Lysate was applied to a 5 mL HisTrap HP column (GE Healthcare Life Sciences), washed with resuspension buffer, and eluted using a linear gradient of 4–100% resuspension buffer + 500 mM imidazole. Proteins were further purified on a Superdex 200 16/60 size exclusion column (GE Healthcare Life Sciences) and transferred to a final storage buffer of 50 mM HEPES pH 8, 100 mM KCl, 15 mM MgCl_2_, 10% glycerol with the addition of 1 mM dithiothreitol for α purifications.

All proteins were judged as purified to homogeneity by sodium dodecyl sulfate polyacrylamide gel electrophoresis (SDS-PAGE) (expected MW 85167 Da for α and 43253 Da for β). Protein concentration was calculated by absorbance at A_280_ (ε = 89050 M^−1^cm^−1^ for α and 61310 M^−1^cm^−1^ for β; coefficients calculated using Benchling). Both α and β were concentrated to 20–30 mg/mL, flash-frozen in liquid nitrogen and stored at −80 °C. A final yield of ~ 20 mg/g cells for α and 8 mg/g cells for β was typical. The specific activities of α and β were measured as 8000 +/− 1000 and 13,000 +/− 2000 nmol dCDP/mg/min, respectively, using a coupled activity assay as previously described (Chen et al., 2018). The coupled assay was completed with *N. gonorrhoeae* thioredoxin (Trx; YP_207791) and thioredoxin reductase (TRR; YP_207723), both codon-optimized and cloned into pET30a(+) vectors and purified using a previously-described protocol for *E. coli* TRR except with 10 mg/L riboflavin added to the TRR growth medium (Zimanyi et al., 2016). The purified *N. gonorrhoeae* TRR contained 13% flavin and concentration was determined from the flavin concentration (ε = 11200 M^−1^cm^−1^ at 458 nm); the *N. gonorrhoeae* Trx concentration was determined using ε = 1490 M^−1^cm^−1^ at 280 nm. The activities of the purified *N. gonorrhoeae* Trx and TRR were determined to be 4900 +/− 300 μmol/(mg-min) and 1830 +/− 50 nmol/(mg-min), respectively, using the micromethod of insulin reduction and DTNB assay, respectively, for activity characterization (Luthman and Holmgren, 1982).

For this structural study, reconstituted and then hydroxyurea-inactivated β2 (met-β2) was used in place of active β2 to increase stability of the protein in storage. Met-β2 was prepared by first reconstituting the diiron cluster and then inactivating the tyrosyl radical as described previously (Ando et al., 2011; Yee et al., 2003). The reconstituted protein contained 89% radical per β2, whereas the hydroxyurea-inactivated protein contained no detectable tyrosyl radical as measured by EPR as described previously (Lin et al., 2017).

To determine iron content in the β2 protein, a ferene assay was used. In an Eppendorf tube, the following components were mixed: 100 μL protein sample (diluted to 20 μM dimer and completed in triplicate) or standard (0–100 μM ferrous ammonium sulfate), 125 μL 0.04% ascorbate solution, 7.5 μL concentrated HCl, 12.5 μL 5 mg/mL ferene (3-(2-pyridyl)-5,6-bis(2-(5-furylsulfonic acid)-1,2,4-triazine) solution. The solution was vortexed for 30 s, and then 500 μL 8 M GuHCl and 100 μL 10 M ammonium acetate were added. The solution was incubated for 30 min at room temperature before the absorbance at 593 nm was measured. Standard curves were calculated from triplicate ferrous ammonium sulfate data, and then protein concentration was calculated using the standard curve. The *N. gonorrhoeae* β used for all experiments contained 1.8 +/− 0.3 iron molecules per monomer.

### 2.2. Cryo-electron microscopy (cryo-EM) specimen preparation and imaging

To prepare cryo-EM specimens, a mix of alpha, beta (1:1.5 ratio α:β), 1 mM dATP, and 1 mM CDP were combined on ice in 50 mM HEPES pH 7.6, 15 mM MgCl_2_, 1 mM EDTA and allowed to incubate for at least one minute before application to the desired grid type (protein concentrations indicated in Table 1). All grids used were copper grids supporting a carbon film. The chameleon-plunged grids were self-wicking grids composed of 1.2 μm holes with 0.8 μm spacing (SPT Labtech) whereas the Vitrobot-plunged grids were C-flat 300 mesh grids with 1.2 μm holes and 1.3 μm spacing (EMS). All grids were glow-discharged prior to use in an easiGlow glow discharger (Pelco). The C-flat grids were glow discharged for 1 min at −15 mA immediately prior to use whereas the chameleon grids were glow discharged at −12 mA for between 60 and 350 s immediately before use; the variation in glow discharge time is necessary to vary the optimal dispense-to plunge time (Supplementary Table 3). The Vitrobot-plunged grids were frozen using a Vitrobot Mark IV (FEI) with a blot force of 5 and a blot time of 5 s with no wait or drain time. chameleon-plunged grids were frozen using the chameleon (SPT Labtech) using the wicking times indicated in Table 1.

All cryo-EM specimens were imaged at 92,000× magnification (resulting in a pixel size of 1.5998 Å on the specimen scale) on a Falcon3EC detector (Thermo Fisher Scientific) in linear mode with underfocus values between 1.2 and 3.1 μm using a Talos Arctica electron microscope (Thermo Fisher Scientific) operating at an accelerating voltage of 200 kV (Supplementary Table 4). Automated data collection was carried out with EPU (Thermo Fisher Scientific); number of exposures ranged from 320 to 620 exposures among the data sets (Supplementary Table 5).

### 2.3. Cryo-electron tomography specimen imaging

Tomography was collected on the Vitrobot-plunged grid and both chameleon 54 ms dispense-to-plunge time grids. Holes were selected to generate a variety of grid square locations in squares with optically similar ice thickness to those squares already collected upon for single particle analysis. All specimens were imaged at 33,000× magnification (resulting in a pixel size of 2.758 Å on the specimen scale) on a K3 detector (Gatan) with an underfocus value of −4.00 μm using a Titan Krios G3i electron microscope (Thermo Fisher Scientific) operating at an accelerating voltage of 200 kV. Automated data collection was carried out with EPU (Thermo Fisher Scientific) using tilt values of −55° to +55° with a 2° step size, 1 fraction per image, and a total exposure on specimen of 86.87 e/Å^2^. Data were collected non-dose-symmetrically with tracking before and after each exposure and focus prior to each acquisition.

### 2.4. Cryo-EM data processing

Cryo-EM data processing was carried out using a combination of Sphire 1.3 (Moriya et al., 2017), Relion 3.0.8 (Scheres, 2012), and Topaz 0.2.3 (Bepler et al., 2019) and is summarized in Supplementary Figures 1 and 2. For each individual data set, individual frames of dose-fractionated exposure were aligned and summed using MotionCor2, and the defocus of the summed frames were estimated using CTER (Penczek et al., 2014; Zheng et al., 2017). The outputs of the MotionCor2 and CTER were used to perform CTF and drift assessments within the SPHIRE software suite (Moriya et al., 2017). The defocus, astigmatism frequency, and drift cutoff values, along with the resulting average drift and final number of micrographs, for each data set are indicated in Supplementary Table 5. An initial set of 500–1000 particles were manually picked for each data set from a subset of aligned movies and were used to train a neural-net automated particle picker using Topaz software. A Topaz cutoff score was chosen to yield 95% recall of true positives (as determined by generation of a precision-recall curve) (Bepler et al., 2019).

Frame alignment and CTF estimation were then rerun on each truncated set of movies using Relion’s implementation of MotionCor2 and CTFFind4, respectively (Mindell and Grigorieff, 2003; Zheng et al., 2017). The coordinate set of all particles in each data set were used to re-extract particles (box size 250 pix) and perform two rounds (25 iterations each) of initial reference-free 2D classification (mask diameter 260 Å) to generate 200 2D class averages. After each round of 2D classification, only intact, ring-shaped classes, which are easy to identify by eye, were selected to continue in the refinement process (see Supplementary Table 6 for the fraction of particles remaining during each step of the refinement process). The particles selected after the second round of 2D classification were used to generate a *de novo* initial reference-free 3D model using no imposed symmetry. Following imposition of D2 symmetry, this model was used for unsupervised 3D classification (6 classes, 100 iterations, no symmetry imposed), and only classes containing intact rings were selected for further refinement in each data set. Following selection of unbroken particles from 3D classes, 7492 particles were randomly selected from each data set for one round of 3D autorefinement. Following refinement, combination of the two half-maps along with B-factor adjustment (“postprocessing”) was performed using a mask generated with a low pass filter of 15 Å, initial binarization threshold 0.0119, binary map extended by 3 pix, with a soft edge of 3 pix. Analysis of 3D FSC results for each data set was completed using the Remote 3DFSC Processing Server found at https://3dfsc.salk.edu/ and these results were analyzed using the two half maps generated from 3D autorefine and the postprocessed map as inputs (Tan et al., 2017).

For processing of the full-size (untruncated) data sets, following selection of particles from 3D classes, all particles in each data set were used during the aforementioned 3D autorefinement and postprocessing steps. Following postprocessing, CTF refinement was performed with CTF parameter fitting and per-particle defocus fitting flagged, and then particle polishing was performed for each data set using the Bayesian method of particle motion estimation in Relion. The mask generated for the chameleon-plunged full size data set was generated with a low pass filter of 15 Å, initial binarization threshold 0.0529, binary map extended by 4 pix, with a soft edge of 3 pix. Similarly, the mask generated for the Vitrobot-plunged full size data set was generated with a low pass filter of 15 Å, initial binarization threshold 0.0089, binary map extended by 3 pix, with a soft edge of 3 pix. The refined particle sets were used to rerun high-resolution 3D refinement and postprocessing with the aforementioned masks to generate the final maps. The final resolution at FSC = 0.143 was 4.3 Å for the chameleon-plunged full size data set and 5.6 Å for the Vitrobot-plunged full size data set. Local resolution was estimated for both data sets using the Relion LocalRes executable within the Relion 3.0.8 GUI.

### 2.5. Cryo-electron tomography processing

Cryo-electron tomography processing was completed using IMOD (Kremer et al., 1996). Briefly, the etomo module was utilized to build, preprocess, align, position, and generate tomograms using a patch-tracking alignment strategy. Positioning and ice thickness determination were done manually based on visual identification of ice boundaries and/or ice contamination at the upper and lower limits of the hole. The 3dmod module was utilized to manually pick particles on those tomograms where particles were visible. Manual picking was done on a 6553 × 6553 Å (2376 × 2376 pixel) box in the center of each hole, matching the dimensions of the micrographs used for single particle analysis. Two independent assessors picked particles to ensure robustness of the resulting relationships. Particles in Supplementary Figure 4 were visualized by collapsing along the y-axis.

### 2.6. Model refinement

Coordinates from the crystal structure of *E. coli* α4β4 RNR bound to CDP and dATP (PDB: 5CNS) were docked into the EM reconstruction of the chameleon-plunged full size data set with Phenix Dock In Map (Adams et al., 2010; Zimanyi et al., 2016). The initial model was adjusted by hand using Coot and further adjusted through iterative refinement by hand in Coot and automatically using Phenix Real Space Refine (Emsley et al., 2010). In real space refinement, resolution was set to 4.0 Å, electron scattering table was selected, NCS constraints for the four αβ subunits were automatically detected and refined, secondary structure hydrogen bonds were relaxed to 0.4 sigma, and secondary structure restraints were manually defined by comparison with PDB 5CNS and with secondary structure restraints that were determined automatically by PHENIX. Definitions for CDP and dATP and the associated Mg^2+^ ions were provided courtesy of Dr. Edward Brignole (Brignole et al., 2018).

Model quality was evaluated using Molprobity (Chen et al., 2010), CaBLAM (Richardson and Richardson, 2013), and EMRinger (Barad et al., 2015) (Supplementary Table 7). The final model contains residues 5 through 738 (of 749) of α, four residues of the histidine tag and residues 1 through 346 and 374 through 376 (of 377) of β, eight dATP molecules, four CDP molecules, six water molecules, two magnesium atoms, and four FeO ligands (Supplementary Table 7). Figures of the model and map were rendered with UCSF Chimera (Pettersen et al., 2004). Coot, Phenix, and Chimera were licensed through the SBGrid Consortium operated out of Harvard Medical School (Morin et al., 2013).

## 3. Results

### 3.1. chameleon-prepared grids with varying dispense-to-plunge times and a control Vitrobot-prepared grid under dATP-inactivating conditions revealed a mixture of intact and dissociated RNR complexes

The chameleon is well positioned as a cryo-EM grid preparation instrument due to the automation of the dispense and plunge steps, the control available in varying dispense-to-plunge time, and the ability to screen grids during freezing. We prepared grids using the chameleon at four different dispense-to-plunge times: 619 ms, 390 ms, 150 ms, and 54 ms. Two different grids were prepared at the 54 ms dispense-to-plunge time, the fastest time available on the instrument, in order to separate grid-to-grid variability from the effects of dispense-to-plunge time. A control grid was also prepared using a Vitrobot with a standard blot time (5 s) and other standard settings (see Methods). The Vitrobot conditions, including blot time, blot force, sample concentration, and buffer conditions, had been extensively optimized, whereas the five chameleon grids were plunged in a two-day period with no prior screening or optimization.

To prepare all cryo-EM specimens, a mix of alpha, beta (1:1.5 ratio α:β), 1 mM dATP, and 1 mM CDP were combined on ice in 50 mM HEPES pH 7.6, 15 mM MgCl_2_, 1 mM EDTA and allowed to incubate for at least one minute before application to the desired grid type (protein concentrations indicated in Table 1). To accurately compare across data sets, collection parameters (microscope, magnification, defocus, camera, approximate dose rate, and exposure time; see Supplementary Table 4) were kept constant across the collection of all data sets. Visual inspection of micrographs from both the chameleon- and Vitrobot-plunged data sets found intact rings as well as rings that were partially or fully dissociated in all data sets (Supplementary Figure 1).

### 3.2. Decreasing dispense-to-plunge time requires an increase in protein concentration to achieve similar particle density on chameleon-prepared grids

Initial screening of chameleon-plunged grids revealed sparse particle distribution that became sparser as dispense-to-plunge time decreased. In order to achieve sufficient particle density for reconstruction, we increased the initial protein concentration from 0.5 mg/mL when using the Vitrobot to 4–6 mg/mL at the fastest chameleon dispense-to-plunge time (Table 1). After automated particle picking using Topaz (95% recall cutoff value), the grid prepared using the chameleon at 54 ms dispense-to-plunge time and 6 mg/mL protein had approximately half as many particles per micrograph than the Vitrobot-prepared grid at 0.5 mg/mL protein (Table 1; Supplementary Figure 3). An alternative method of counting particles by two independent researchers using tomography corroborated that higher concentrations of initial sample led to similar but still slightly lower (0.55 – 0.86x) concentrations of particles within holes for fast-plunged chameleon grids versus the Vitrobot-plunged grid even with the very different concentrations of sample applied to these grids (Supplementary Figure 4; Supplementary Movies 1 and 2). A clear decrease in picked particles per micrograph was also seen in the chameleon grids prepared at 1 mg/mL protein and 619, 390, and 150 ms dispense-to-plunge times, respectively. Additionally, even at a relatively slow (619 ms) dispense-to-plunge time, approximately twice the sample concentration was needed for chameleon plunging compared to Vitrobot plunging to achieve a similar number of particles per micrograph.

The number of particles seen per micrograph on chameleon-plunged grids was closer to the number of particles we would expect based on initial protein concentration, field of view, and estimated ice thickness. Although there is likely some undercounting when using the 2D images of both Vitrobot-plunged and chameleon-plunged grids due to particle aggregation or stacking on top of one another, the metric of average particles picked per micrograph is more robust than particles picked via tomography. This robustness is due to the higher numbers of micrographs and the ability to directly use the micrographs that contributed to the final reconstructions, as opposed to the tomography that was necessarily performed on a much small number of holes. The amount of particle stacking observed between chameleon-plunged and Vitrobot-plunged grids was similar as seen in the tomogram representations in Supplementary Figure 4. The tomographic data also demonstrated a trend of particle number variation by location within the square (Supplementary Figure 4). Thus, grids plunged using the chameleon may be providing a more accurate reflection of how particles behave in solution at a given concentration and buffer conditions than the Vitrobot, in which we observed a concentration effect of more than 200x on the grids as opposed to solution, likely due to the concentrating effects of blotting or evaporation (Table 1). However, other causes of variation in particle concentration during the dispense and freeze steps, such as shearing effects due to self-wicking grids and adsorption/desorption effects, are also at play and were not investigated in this study. We additionally note that both the fast plunged chameleon and the Vitrobot-plunged grids had particles primarily aligned at the air/water interface; however, the chameleon grids had more particles on both the top and bottom ice surfaces, whereas the Vitrobot-plunged grids primarily had particles only on one surface (Supplementary Figure 4; Supplementary Movies 1 and 2).

### 3.3. Decreasing dispense-to-plunge time decreases ring denaturation for the N. gonorrhoeae RNR

**Fig. 1.**
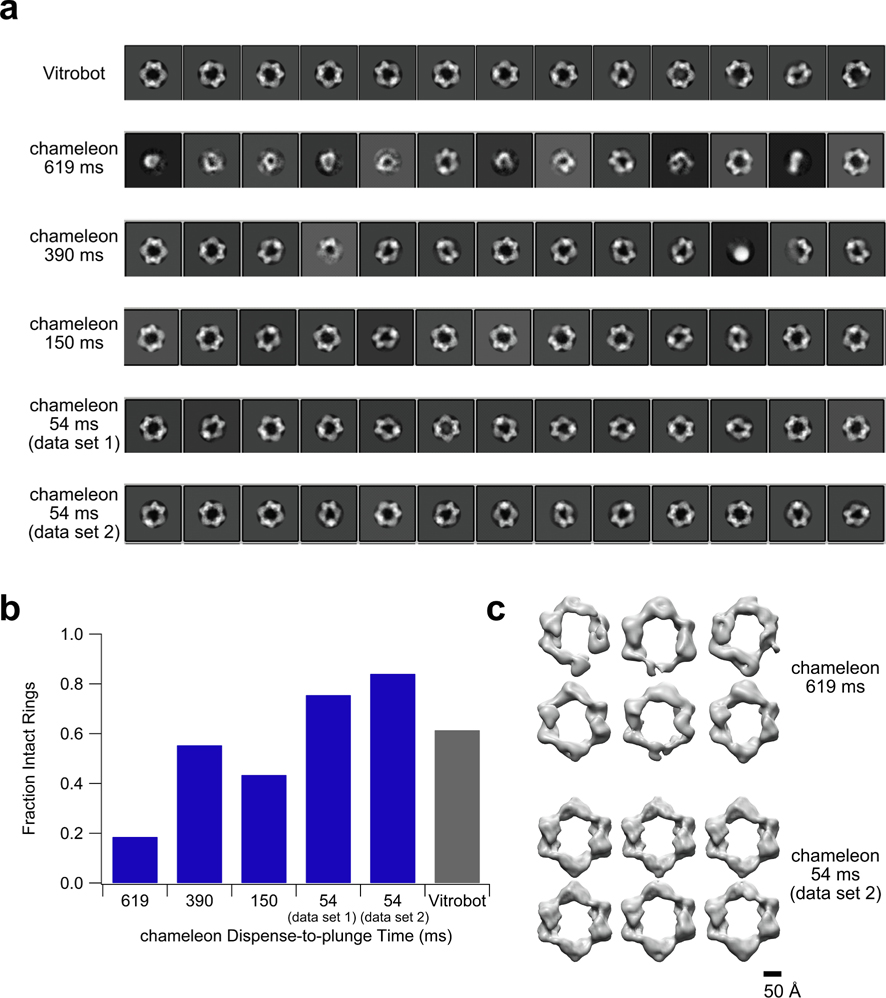
Decreasing chameleon dispense-to-plunge time results in a smaller amount of particle denaturation. (a) The top thirteen 2D classes for each data set taken in this study. The total number of 2D classes calculated for each sample was 200. (b) The fraction of particles contributing to 2D classes that contained intact rings versus the total number of particles in classes containing intact or broken rings for each data set taken in this study. Ambiguous classes (i.e. side views where the intact state of the particles could not be determined) and non-ring-shaped classes were excluded from the analysis. (c) 3D classes for the chameleon 619 ms and 54 ms (data set 2) dispense-to-plunge time data sets. Six classes and 100 rounds of classification were specified for each data set. Particle inputs to the 3D classification were those that contributed to 2D classes consisting of intact rings or ambiguous side views in each of two rounds of 2D classification.

The increase in protein concentration that is needed to create comparably dense grids when using faster chameleon dispense-to-plunge times emphasizes that the extent of evaporation and protein interaction with the air/water interface varies for different dispense-to-plunge times. We first analyzed whether these variations would result in differences in the extent of particle denaturation seen for the 528-kDa *N. gonorrhoeae* RNR that forms rings under the chosen (dATP inhibiting) conditions. For ring-shaped structures such as the complex being studied, it is relatively straightforward to coarsely determine by eye whether or not the complex is intact or partially denatured by looking for the presence of an intact or broken ring in top or bottom views of the complex. After 2D classification, the 2D classes from grids plunged using faster chameleon dispense-to-plunge times contained fewer broken rings than those from grids plunged using slower chameleon dispense-to-plunge times (Fig. 1a). Quantification of the number of particles present in 2D classes consisting of intact rings versus the total number of particles present in 2D classes revealed a trend towards a larger fraction of intact rings for grids prepared using a faster dispense-to-plunge time (Fig. 1b, Supplementary Figure 5). At the fastest dispense-to-plunge time of 54 ms, the fraction of intact rings surpassed that seen on the Vitrobot-prepared grid. Additionally, even after two rounds of 2D classification and selection for only classes containing intact rings, 3D classification revealed that grids prepared with the Vitrobot or with the chameleon using longer dispense-to-plunge times still had numerous partially denatured particles present within the “intact” 2D classes (Fig. 1c, Supplementary Figure 6). Notably, the fraction of intact rings does not correlate exactly with dispense-to-plunge time, and the two 54 ms data sets vary slightly, indicating that other factors, such as ice thickness, shroud humidity, or time since sample aspiration may also impact particle denaturation (Supplementary Figure 7, Supplementary Table 8). The 54 ms dispense-to-plunge time data set 1 had thinner ice (37 ± 9 and 60 ± 10 nm in the center and edge of holes, respectively; n = 11 holes) as compared to the 54 ms dispense-to-plunge time data set 2 (44 ± 6 and 67 ± 9 nm; n = 7 holes) as measured by tomography (Supplementary Table 9).

### 3.4. Preferred orientation is not affected by dispense-to-plunge time for the complex and particles studied

**Fig. 2.**
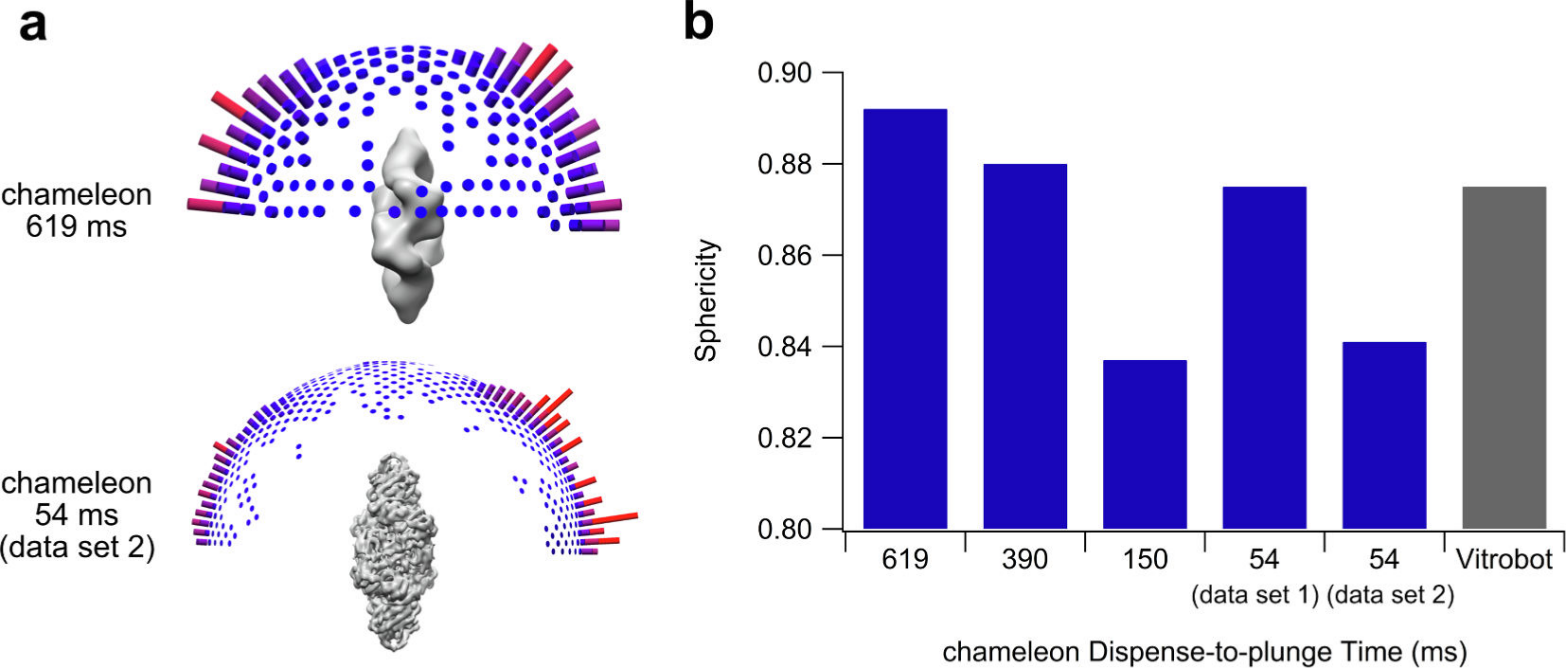
Chameleon dispense-to-plunge time does not affect preferred orientation of the RNR sample. (a) Angular distribution plots visualized in Chimera generated from assigned particle views during the Relion 3D autorefine process. The width of the cylinders varies due to the use of different angular sampling parameters for lower resolution (619 ms dispense-to-plunge) versus higher resolution (54 ms-dispense-to-plunge) reconstructions. A larger or redder cylinder indicates more particles contributing to that view of the reconstruction. Each map was generated through the Relion 3D autorefine and postprocess steps with 7492 particles and is shown at an RMSD value of 6. (b) Calculated half map sphericity values for each data set. Sphericity values were calculated using the 3DFSC server (3Dfsc.salk.edu) and can range from 0 (minimum, only one orientation visible) to 1 (maximum, all views equally represented) (Tan et al., 2017).

In addition to particle denaturation limiting the quality of a cryo-EM single particle reconstruction, preferred orientation of particles on a cryo-EM grid can both generate reconstruction artifacts and limit the final resolution of a structure due to a lack of views of one or more regions of the particle. Ring-shaped particles such as the RNR complex studied here commonly display preferred orientation due to a propensity to fall flat on the grid. Thus, we qualitatively and quantitatively assessed whether preferred orientation of our RNR complex was affected by varying dispense-to-plunge times of the chameleon. In order to accurately compare between data sets, all data sets were refined with the same number of final particles (7492 particles), selected randomly from the output of the chosen 3D classification step. The number of particles used was dictated by the number of final particles in the limiting data set (150 ms dispense-to-plunge time) (Supplementary Table 6). We found no effect of dispense-to-plunge time or plunging method (chameleon versus Vitrobot) on preferred orientation of this sample when measured using angular distribution plots or half map sphericity values (Fig. 2, Supplementary Figure 8) (Tan et al., 2017). We do note that all calculated half map sphericity values are above 0.8, indicating minimal to no artifacts were seen in model data (Tan et al., 2017).

### 3.5. Final resolution of the N. gonorrhoeae RNR structure improves with decreased dispense-to-plunge time

**Fig. 3.**
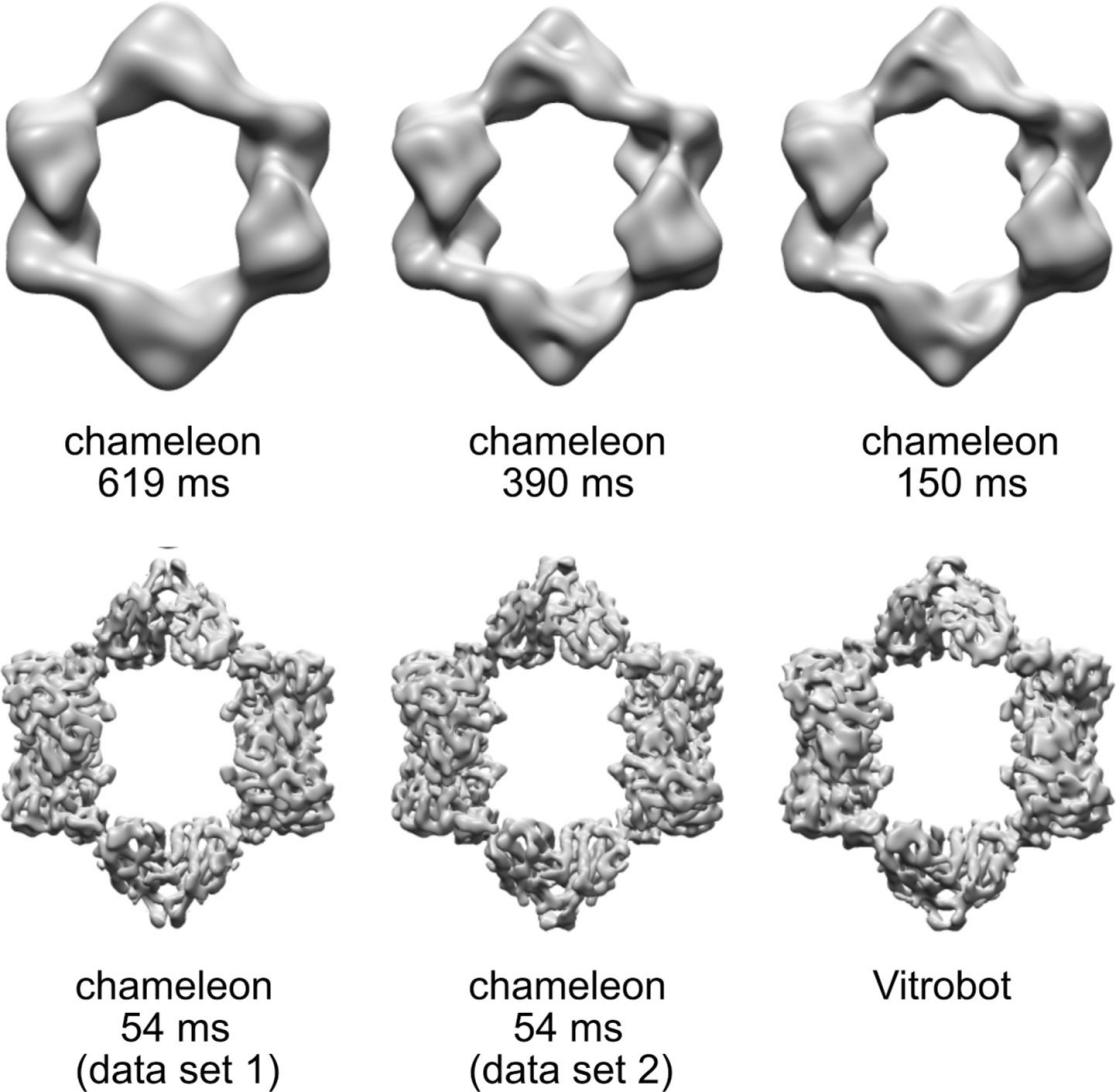
Decreasing chameleon dispense-to-plunge time increases resolution of the final reconstruction. The plunging instrument and dispense-to-plunge time (where applicable) are indicated below each structure. Each structure was generated using 7942 particles randomly selected from all particles remaining after the 3D classification step. All structures are shown after postprocessing in Relion at an RMSD value of 6. The final resolutions of each data set are 23 Å, 21 Å, 18 Å, 10 Å, 7.3 Å, and 8.7 Å resolution, reading left to right, top to bottom across the figure.

The ultimate goal of most single particle cryo-EM studies is to produce a high-resolution structure of the protein or complex of interest. Thus, we investigated whether the trend seen between dispense-to-plunge time and extent of particle denaturation carried over to the final resolution of the reconstructions generated from 7942 particles. Although the number of particles in each reconstruction is insufficient to produce a high-resolution reconstruction, we saw a clear trend in increasing resolution of structures as dispense-to-plunge time of the grid decreased (Fig. 3). At this particle-number-limited resolution, we do not see any notable differences between the reconstructions generated using particles from the Vitrobot-prepared grid and the fastest dispense-to-plunge time chameleon-prepared grids.

### 3.6. In untruncated data sets, a chameleon-plunged grid processes to a higher resolution than the Vitrobot-plunged grid and processes to the functional resolution limit for the image pixel size

**Fig. 4.**
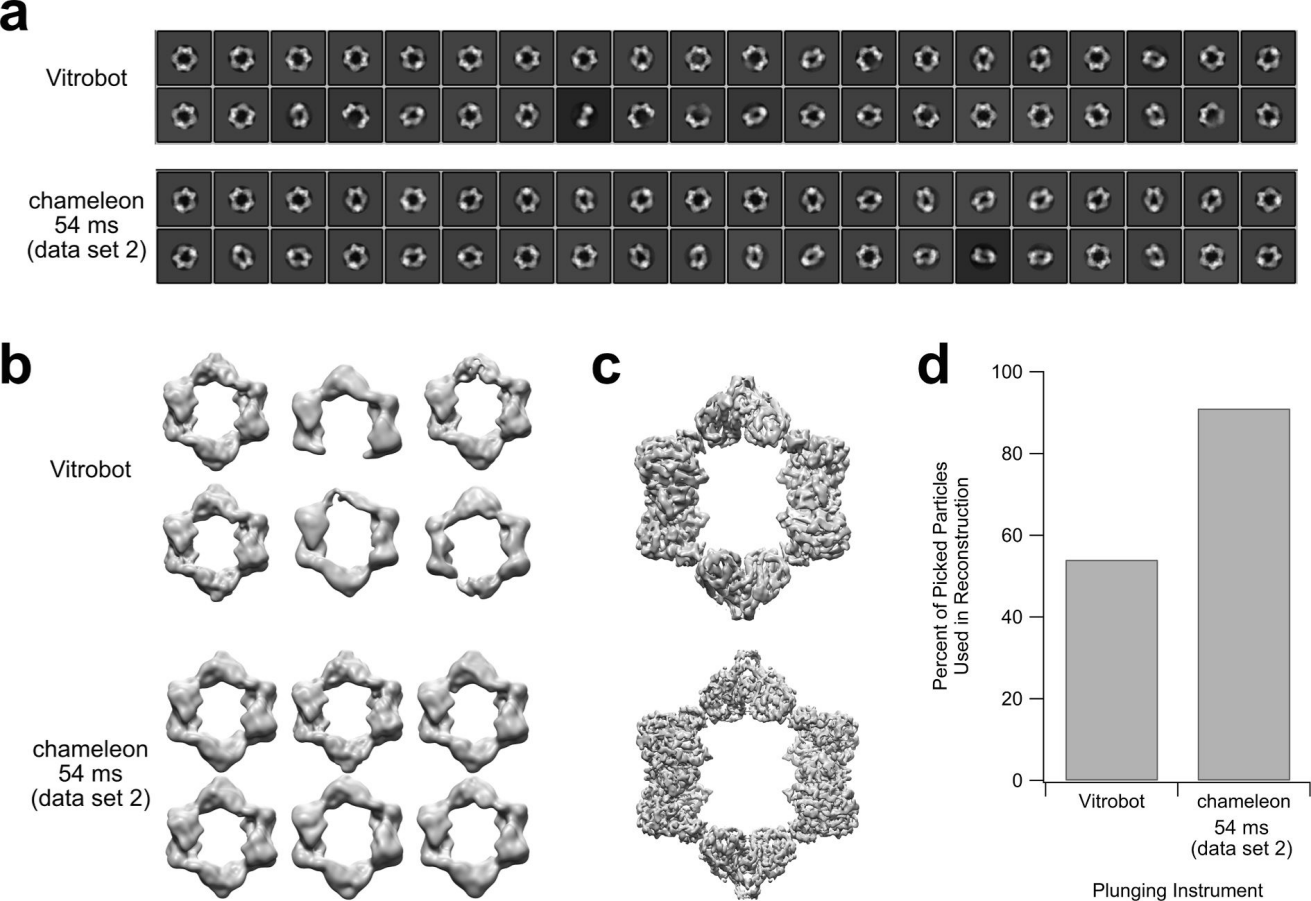
A chameleon grid plunged using a 54 ms dispense-to-plunge time produced a data set that processes better and more efficiently than a data set produced from a Vitrobot-plunged grid. (a) Top 40 2D classes (of 200 total classes) of data sets from a Vitrobot-plunged grid and a chameleon-plunged grid. 2D classes were generated using 25 iterations in Relion. (b) 3D classes generated from the Vitrobot-plunged grid and the chameleon-plunged grid. Six classes and 100 rounds of classification were specified in Relion for each data set. Particle inputs to the 3D classification were those that contributed to 2D classes consisting of intact rings or ambiguous side views in each of two rounds of 2D classification. All classes are visualized at an RMSD value of 6. (c) Postprocessed reconstructions of the data from the Vitrobot-plunged grid (top) and a chameleon-plunged grid (bottom). Reconstructions are at 5.6 Å and 4.3 Å resolution, respectively. Both reconstructions are visualized at an RMSD value of 6. (d) Comparison of the percentage of initially-picked particles (at 95% recall in Topaz) that were used to generate the final reconstruction shown in (c) for the data sets generated from the Vitrobot-plunged grid and the chameleon-plunged grid.

The current limited availability and novelty of the method associated with use of the chameleon versus standard plungers such as the Vitrobot can dissuade new users from expanding their plunging repertoire. Thus, we further probed whether the best chameleon-plunged data set processed comparably, better, or worse than our Vitrobot-plunged data set. The sample buffer and plunging conditions used with the Vitrobot in this study were determined by optimizing multiple detergents, additives, blot times, blot forces, and grid manufacturers and hole spacings in order to generate an optimal Vitrobot-plunged data set (data not shown). The chameleon-plunged data set used for further analysis was the most promising dataset identified above (54 ms data set 2) when truncated to 7492 particles. With all particles present (56,614 and 50,425 particles for the Vitrobot- and chameleon-plunged data sets, respectively), the chameleon-plunged data set showed more intact rings and higher quality reconstructions throughout the 2D classification and 3D classification steps as well as through 3D autorefinement and final postprocessing (Fig. 4a–c; Supplementary Fig. 2).

The Vitrobot-plunged data set processed to 5.6 Å resolution, whereas the chameleon-plunged data set processed to 4.3 Å resolution (Fig. 4c). The two reconstructions had similar local resolution distributions, indicating that the extent of air/water interface denaturation is likely influencing final resolution more than any physical effects associated with blotting versus spraying of sample (Supplementary Figure 9). We note that with the 1.5998 Å/pixel magnification used during collection, we are approaching the resolution limit (2x the pixel size) with the chameleon-plunged data set but not the Vitrobot-plunged data set, indicating that the chameleon-plunged data set could increase in resolution if a smaller pixel size were used during data collection. Notably, an unintended consequence of sample concentration changes was that the chameleon-plunged sample contained a much higher concentration of glycerol than the Vitrobot-plunged sample (3.06% glycerol in the 6 mg/mL sample vs 0.19% glycerol in the 0.5 mg/mL sample, respectively). However, a Vitrobot-plunged sample at 0.5 mg/mL protein and 3.06% glycerol, mimicking the chameleon-plunged sample conditions, generated a worse data set (8.3 Å resolution) when compared to the Vitrobot-plunged sample with a lower percentage of glycerol, indicating glycerol variability was not the source of the higher quality data seen in the chameleon sample (Supplementary Figure 10).

We also noted that the larger fraction of intact complexes present in the chameleon-plunged data set versus the Vitrobot-plunged data set led to a substantially higher percentage of picked particles being used in the final reconstruction (91% vs 54%, respectively) (Fig. 4d; Supplementary Table 6). This finding could have implications for shorter collection times and more efficient use of microscope time, which is often a limiting factor for scientists; combined with the pre-screening of grids during the chameleon freezing process, chameleon plunging could lead to a significant decrease in microscope time and expense for scientists.

### 3.7. The data set generated from a chameleon-plunged grid provides biological insight into the structure of the inactive N. gonorrhoeae RNR complex

**Fig. 5.**
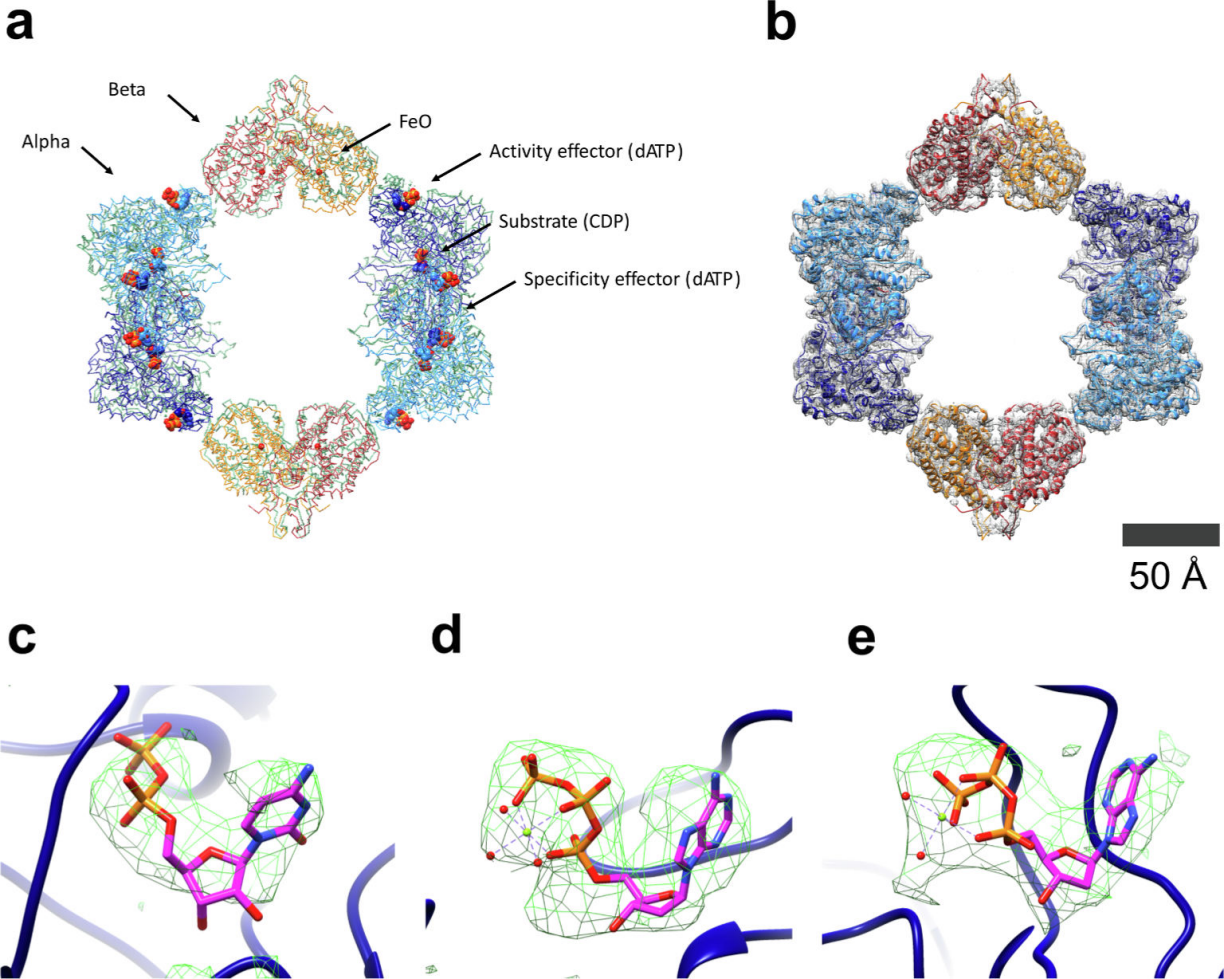
The chameleon-plunged grid yielded a structure of the *N. gonorrhoeae* RNR with implications for biology and drug development. (a) A comparison between the inactive structures of the *N. gonorrhoeae* class Ia RNR (blue/orange) and the *E. coli* class Ia RNR (green; PDB 5CNS) (Zimanyi et al., 2016). Bound ligands in a single alpha and beta monomer are labeled. (b) The *N. gonorrhoeae* model fit into the calculated density (at 6σ) from the data set generated from the chameleon-plunged grid. (c) Difference density for the CDP substrate in the substrate-binding pocket. (d) Difference density for the dATP allosteric activity effector. (e) Difference density for the dATP allosteric specificity effector. All difference density was calculated using Phenix.real_space_diff_map with ligands removed from the PDB and is contoured at 6σ.

Although the data set generated from the chameleon-plunged grid was limited to 4.3 Å resolution, we were still able to elucidate previously-unknown structural features of the *N. gonorrhoeae* RNR. First, we confirmed that in the presence of activity effector dATP, the *N. gonorrhoeae* class Ia RNR forms a protein complex similar in structure and subunit composition to the well studied *E. coli* class Ia RNR. In particular, when dATP is bound to the activity sites within the N-terminal regions (i.e., cone domains) of either the *E. coli* or *N. gonorrhoeae* α subunits, each α subunit interacts with a β subunit, forming four α/β interfaces and an α4β4 ring-shaped structure (Fig. 5a,b) (Ando et al., 2011; Zimanyi et al., 2016, 2012). Within the *N. gonorrhoeae* α/β interface, there is density for residues homologous to the *E. coli* class Ia RNR interface residues, indicating close similarity between the two structures in this region (Supplementary Figure 11a); additionally, a similar amount of solvent-accessible surface area (495 Å^2^ and 575 Å^2^ for *N. gonorrhoeae* and *E. coli*, respectively) is buried in the α/β interface upon formation of the α4β4 structure. Density for both residues S41 and H25, which have recently been identified as important residues for organism viability and drug resistance, are visible in the α/β interface region, suggesting that the compounds may act on the α/β interface region (Supplementary Figure 11a) (Narasimhan et al., 2020). Clear density is also visible for the ribonucleotide substrate and effectors bound in the structure, with the expected dATP molecules present as both activity and specificity effector and CDP present as substrate (Fig. 5c–e). The similarities highlighted here indicate that many of the findings of the decades of study on the *E. coli* class Ia RNR can be used as starting points for study of the *N. gonorrhoeae* RNR, providing many testable hypotheses for a medically-relevant RNR.

In addition to the similarities between the *N. gonorrhoeae* and *E. coli* class Ia RNR structures, the new structure also reveals a clear difference between the *N. gonorrhoeae* and *E. coli* RNR inactive structures in the region of the β subunit tail residues (Supplementary Figure 11b). In contrast to the *E. coli* structure, in which an entire helix comprised of tail residues from the β subunit reaches around to each α subunit in the inactive structure, the *N. gonorrhoeae* structure has only three residues visible in the cryo-EM structure, indicating much more disorder in the β tail regions in the inactive state of *N. gonorrhoeae* versus *E. coli* class Ia RNR. Future structural studies of this complex at a smaller pixel size using chameleon 54 ms dispense-to-plunge times have the potential to yield further molecular insights into this medically-relevant protein.

## 4. Discussion

The development of the chameleon as a next-generation, blot-free grid preparation instrument with commercially available compatible grids is a new step in tackling persistent sample preparation roadblocks in the field of cryo-EM (Jain et al., 2012; Razinkov et al., 2016; Wei et al., 2018). Here, we were able to generate a structure of the *N. gonorrhoeae* inactive RNR complex resolved to 0.744x the Nyquist limit (4.3 Å resolution at 1.5998 Å/pixel) through the use of the blot-free chameleon with a fast (54 ms) dispense-to-plunge time but not through a traditional blotting-based plunging technique (which produced a 5.6 Å resolution structure) (Fig. 4). We anticipate that a higher resolution structure of this complex can be obtained using the chameleon to plunge and a smaller pixel size during a future collection. We additionally found that for this sample, decreasing dispense-to-plunge time on the chameleon led to a decrease in particle denaturation on the grids (Fig. 1). We used tomography to investigate whether the decrease in particle denaturation was due to fewer particles clustered at the air/water interface and found that particles did still cluster at the air/water interface in chameleon-plunged grids. However, faster plunge times and the absence of blotting paper could be limiting the number of interactions per particle at the air/water and blotting paper (where relevant) interfaces. Particles could also be spending less time adsorbed to the air/water interface, leading to decreased particle denaturation. Regardless of the exact mechanism that accounts for decreased particle denaturation, our results emphasize the importance of screening dispense-to-plunge time parameters in addition to screening plunging method (Armstrong et al., 2020; Klebl et al., 2020; Noble et al., 2018a,b). We note that there is potentially more particle aggregation on the chameleon fast plunge grids than the Vitrobot-plunged grid. This difference could be due to more overlapping particles as a result of particles being present at both air/water interfaces instead of just one interface (Supplementary Figure 4 and Supplementary Movies 1 and 2). This particle overlap did not affect the quality of the reconstruction and highly overlapping particles were effectively omitted from both Vitrobot and chameleon data sets by using the 95% recall function in Topaz.

Determination of sample and plunging conditions needed to produce high-quality EM grids is highly variable by sample and based largely on trial-and-error (Reviewed in Drulyte et al., 2018); although results for the chameleon can also vary by sample, our study demonstrates that variation in plunging method and dispense-to-plunge time should be a first line of optimization in sample preparation. We additionally demonstrate that large data sets are unnecessary in order to screen for improvement using different sample preparation techniques, and that sample improvements can be visualized and quantified as early as the 2D classification step (Fig. 1). Although we saw no improvements in preferred orientation for our sample based on plunging instrument or dispense-to-plunge time, we note that others have observed this effect (Dandey et al., 2018; Klebl et al., 2020; Noble et al., 2018a,b; Tan et al., 2017), and that this sample did not display enough preferred orientation as measured by the map-to-model sphericity value to cause distortions in the final reconstructions. Additional benefits to using blotless plunging techniques, such as the elimination of sample contamination by carryover on blot pads, can also be considered when choosing plunging method. Based on our current work where the two 54 ms dispense-to-plunge time data sets did not process to the same resolution, we also suggest that other variables, such as ice thickness and chamber humidity, should be investigated and incorporated into the screening process. The first 54 ms dispense-to-plunge data set, which processed to a worse resolution than the second 54 ms dispense-to-plunge data set, had a smaller minimum ice thickness as measured by tomography (270 vs 390 Å, respectively), suggesting that the 210 Å diameter particles require slightly thicker ice than 270 Å for optimal imaging and reconstruction. However, because the tilt series were collected on similar, nearby squares rather than directly from squares where the 2D micrographs were collected, we are cautious about drawing conclusions without further study.

In agreement with results from other spray-based sample preparation techniques, we note that there was an increase in sample concentration needed to produce the same number of particles per micrograph when using the chameleon versus a standard blotting device, and that the effect was exacerbated with decreasing chameleon dispense-to-plunge times (Dandey et al., 2018; Klebl et al., 2020; Kontziampasis et al., 2019). We found that faster dispense-to-plunge times generate grids that have particles more closely matching the initial concentration of the sample, potentially due to less evaporation, diffusion effects on the nanowire grids, limiting adsorption to the air/water interface, and no concentrating effects of the blotting paper (Table 1) (Glaeser, 2018). For researchers studying biological samples under physiological conditions, the control over buffer and sample concentration during the sample preparation process in fast plunge chameleon grids may be an important consideration for production of physiologically-relevant structures. The higher starting concentration may also push the equilibrium of some samples towards complexes versus their individual protein components, further allowing for more intact complexes to be seen on fast plunged chameleon grids. We additionally note that ice thickness varies systematically across chameleon-plunged holes, with thinner ice in the middle than at the edge of the holes, and also by location of the holes in the grid square, with thinner ice towards the outside of the grid squares due to the effects of wicking. These observations may aid future researchers in determining where and how to image their chameleon-plunged grids.

We were able to utilize the chameleon to determine the structure of the dATP-inactivated state of class Ia RNR from *N. gonorrhoeae* previously unsolved at a molecular resolution (Fig. 5). The determination that dATP-inactivated *N. gonorrhoeae* RNR forms an α4β4 structure that is notably different from the human RNR dATP-inactivated α6 state opens doors for future drug design targeting the *N. gonorrhoeae* RNR inactive state because no cross-reactivity is expected against the human enzyme. Drugs that stabilize the inactive state of RNR have already been shown to be effective as anticancer drugs (Aye et al., 2012; Aye and Stubbe, 2011), and so we anticipate similar success for newly designed or discovered *N. gonorrhoeae* inhibitors that stabilize the inactive α4β4 state, including compounds we recently reported that may inhibit *N. gonorrhoeae* RNR via the α/β interface (Aye et al., 2012; Aye and Stubbe, 2011; Narasimhan et al., 2020). Atomic resolution structures will aid in further drug design and interpretation of existing biochemical and cross-reactivity data.

Together, our study suggests that the fast dispense-to-plunge time afforded by the blot-free sample preparation method of the chameleon is invaluable for increasing particle quality for some cryo-EM protein samples. This improvement in particle quality is particularly true for proteins such as the *N. gonorrhoeae* RNR complex studied here, which forms ring-shaped oligomers that can easily dissociate upon contact with the air/water interface or upon other physical or biochemical stressors. We anticipate that the chameleon and other blot-free sample preparation instruments, particularly at fast dispense-to-plunge times, will become a standard part of the screening process and will prove themselves necessary for generation of high-quality data sets for difficult EM samples, including for further studies of the *N. gonorrhoeae* and other bacterial RNRs necessary for furthering biochemical analysis and drug design.

## Supplementary Material

Supplemental data1

Supplemental Movie 1

Supplemental Movie 2

## Figures and Tables

**Table 1:** Comparison of sample concentration and resulting average particles per micrograph for six different cryo-EM grids prepared using the chameleon at different dispense-to-plunge times or the Vitrobot. Particle picking was completed using Topaz after manual picking of approximately 1000 particles and a cutoff value calculated to produce 95% recall using a precision-recall curve (Bepler et al., 2019). Theoretical particles per micrograph values were calculated using the known field of view and magnification of the microscope and an ice thickness ranging from 28 nm (minimum) to 76 nm (maximum) for chameleon grids and 39 nm (minimum) to 55 nm (maximum) for Vitrobot grids along with the known protein concentration. The range of ice thicknesses were determined by using the average hole center ice thickness − 1 SD (min) and the average hole edge ice thickness + 1 SD (max) as determined by tomography (Supplementary Table 9).

Plunging method	Wicking time (ms)	Protein concentration (mg/mL)	Particles per micrograph (observed, 95% recall)	Particles per micrograph (theoretical)	Ratio (observed particles / theoretical particles)

Vitrobot	n/a	0.5	208	1	208
chameleon	619	1	175	14–37	13–5
	390	1	269	14–37	19–7
	150	1	74	14–37	5–2
	54	4	71	55–149	1–0.5
	54	6	95	82–223	1–0.4
