# Supplementary material for: Effects of chameleon dispense-to-plunge speed on particle concentration, complex formation, and final resolution: A case study using the Neisseria gonorrhoeae ribonucleotide reductase inactive complex
Source: J Struct Biol. Author manuscript; Available in PMC 2022 Apr 9. (PMC8994553; doi:10.1016/j.jsb.2021.107825)
Supplement: Supplemental data1 [file NIHMS1789070-supplement-Supplemental_data1.pdf]

Supplementary Material for

Effects of chameleon dispense-to-plunge speed on particle concentration, complex formation, and final resolution: A case study using the *Neisseria gonorrhoeae* ribonucleotide reductase inactive complex

Talya S. Levitz[a], Edward J. Brignole[a, b], Ivan Fong[c], Michele C. Darrow[c, *], Catherine L. Drennan[a, d, e, *]

[a] *Department of Biology, Massachusetts Institute of Technology, 77 Massachusetts Avenue, Cambridge, MA, USA*
[b] *MIT.nano, Massachusetts Institute of Technology, 77 Massachusetts Avenue, Cambridge, MA, USA*
[c] *SPT Labtech Melbourn Science Park, Cambridge Rd, Melbourn SG8 6HB, United Kingdom*
[d] *Department of Chemistry, Massachusetts Institute of Technology, 77 Massachusetts Avenue, Cambridge, MA, USA*
[e] *Howard Hughes Medical Institute, Massachusetts Institute of Technology, Cambridge, MA USA*

* Corresponding authors at: michele.darrow@sptlabtech.com (M.C. Darrow), cdrennan@mit.edu (C.L. Drennan).

Supplementary Movie 1. Cryo-electron tomography on a representative hole of the fast plunged chameleon grid (data set 2). A representative tomogram is shown as consecutive slices along the z-axis, first with no particles picked and then subsequently with all particles picked for this sample. Particles were picked in a 2376x2376 pixel (6553 x 6553 Å box) in the middle of the hole to model the micrograph size and location used in the cryo-electron microscopy experiments. Particle picking and tomogram reconstruction were completed using IMOD (Kremer et al., 1996).

Supplementary Movie 2. Cryo-electron tomography on a representative hole of the Vitrobot-plunged grid. A representative tomogram is shown as consecutive slices along the z-axis, first with no particles picked and then subsequently with all particles picked for this sample. Particles were picked in a 2376x2376 pixel (6553 x 6553 Å box) in the middle of the hole to model the micrograph size and location used in the cryo-electron microscopy experiments. Particle picking and tomogram reconstruction were completed using IMOD (Kremer et al., 1996).

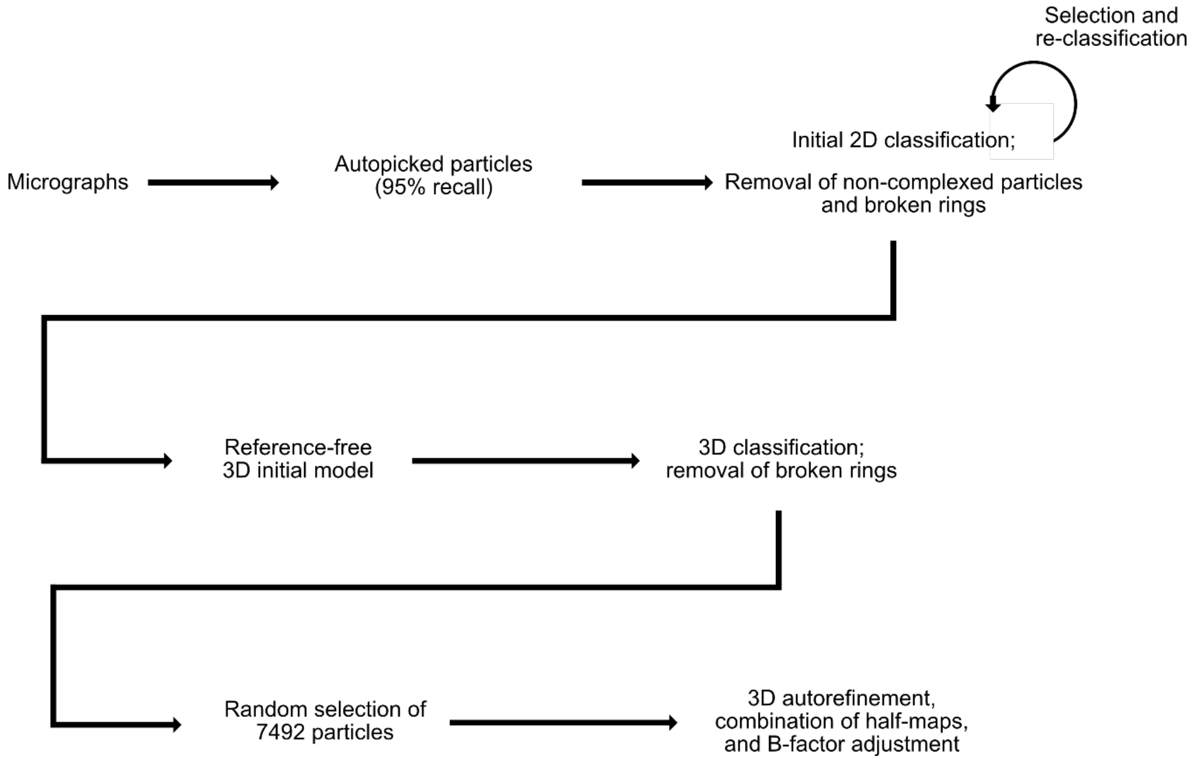

Supplementary Figure 1. Workflow for truncated cryo-EM data set processing. Drift assessment and initial CTF assessment for micrograph selection was completed using SPHIRE (Moriya et al., 2017), particles were autopicked using Topaz (Bepler et al., 2019), and all subsequent analysis was performed using Relion 3.0 (Scheres, 2012).

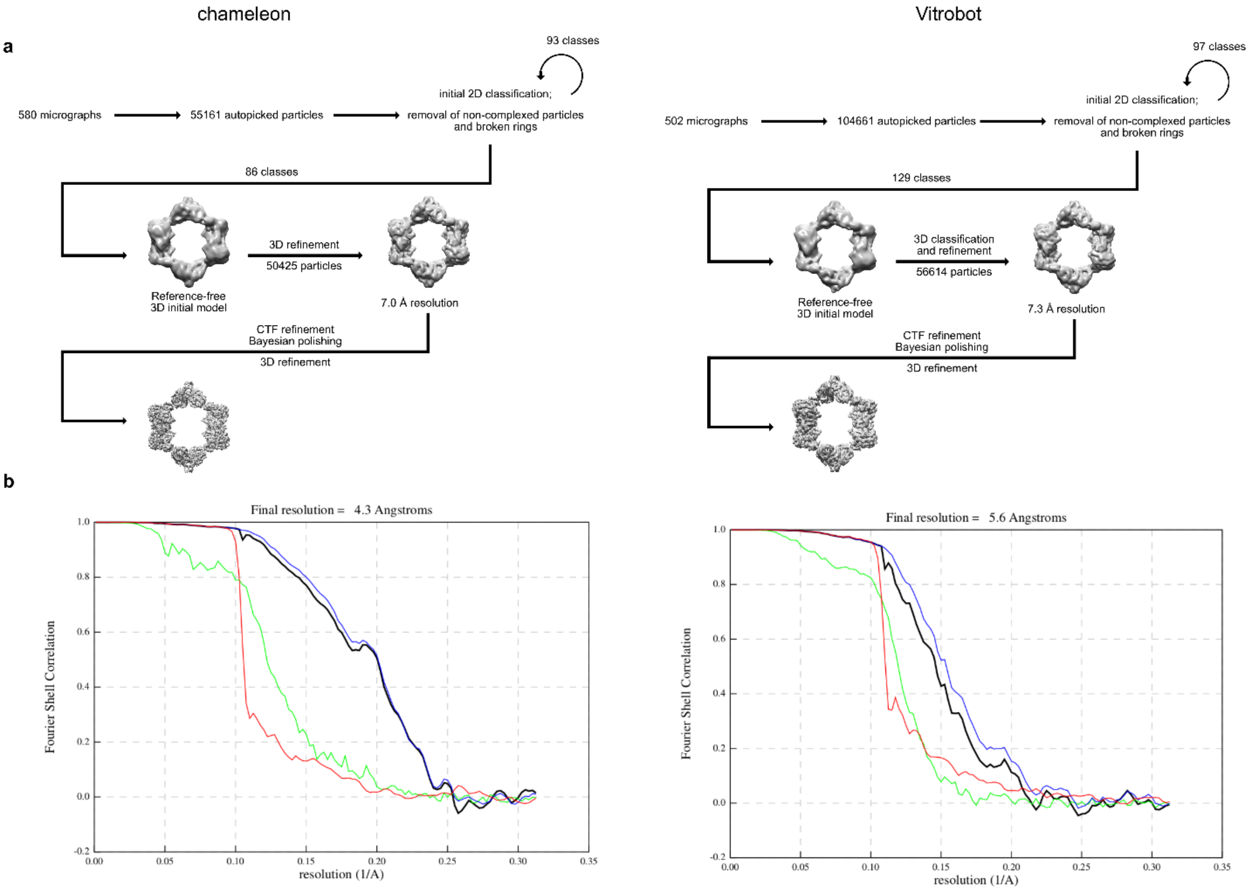

Supplementary Figure 2. Workflow and FSC curves of full-sized data set cryo-EM data processing. (a) Data processing workflow for the untruncated chameleon (left) and Vitrobot (right) data sets. Drift assessment and initial CTF assessment for micrograph selection were completed using SPHIRE (Moriya et al., 2017), particles were autopicked using Topaz (Bepler et al., 2019), and all subsequent analysis was performed using Relion 3.0 (Scheres, 2012). (b) FSC plots for corrected (black), unmasked (green), masked (blue), and phase-randomized masked (red) maps for chameleon (left) and Vitrobot (right) reconstructions shown in (a). Resolution indicated at the top of each figure is the resolution at 0.143 FSC on the corrected map. Plots in (b) were generated using Relion 3.0 (Scheres, 2012).

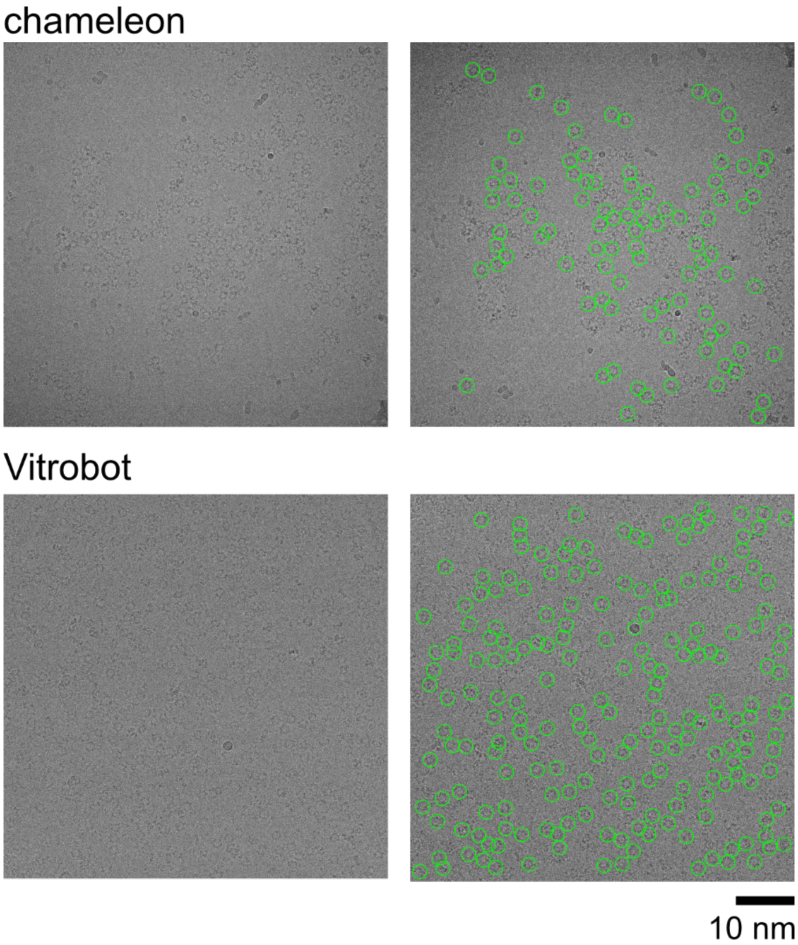

Supplementary Figure 3. Representative micrographs and picked particles after imposition of the 95% recall cutoff. Micrograph from a chameleon-plunged grid (54 ms dispense-to-plunge time; data set 2; top) and the Vitrobot-plunged grid (bottom) with the 93 and 201 picked particles circled in green, respectively. Picked particles were visualized using Relion’s ManualPick interface.

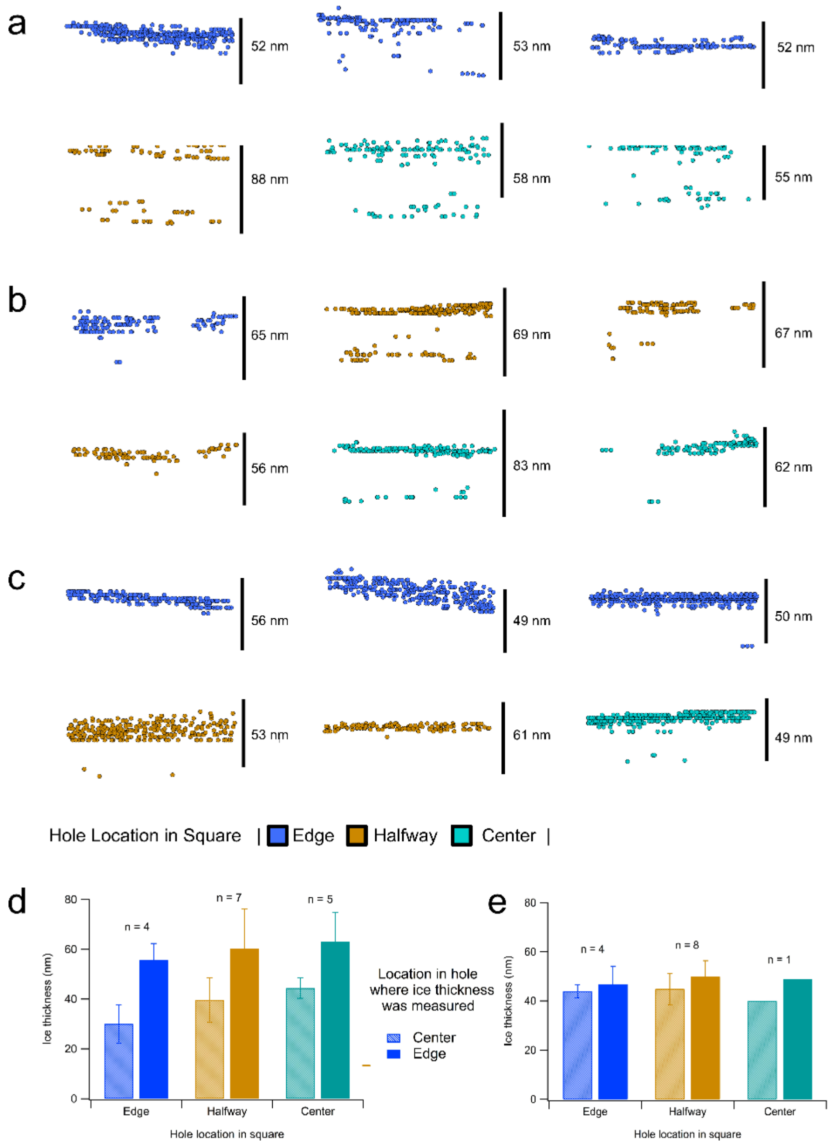

Supplementary Figure 4. Tomography of the Vitrobot-plunged grid and the two fast-plunge chameleon grids. (a) Particle distributions within the ice of the 54 ms data set 1 from a chameleon-plunged grid. (b) Particle distributions within the ice of the 54 ms data set 2 from a chameleon-plunged grid. (c) Particle distributions within the ice of the Vitrobot-plunged grid. All scale bars indicate the ice thickness measured at the edge of the hole. (d) Average ice thicknesses of the center (stripes) and edge (solid) of holes found at the edge (blue), halfway (orange), or center (teal) of grid squares in two fast-plunged chameleon grids. For location in grid square, the ratio of center hole thickness to edge hole thickness was 1 : 1.1-1.5 whereas the ratio of halfway hole thickness to edge hole thickness was 1 : 1.1-1.3 (range indicates variation in comparing thickness of hole edges versus hole centers). (e) Average ice thicknesses of holes from the Vitrobot-plunged grid. For location in grid square, the ratio of center hole thickness to edge hole thickness was 1 : 0.9-1.0 whereas the ratio of halfway hole thickness to edge hole thickness was 1 : 1.0-1.1 (range indicates variation in comparing thickness of hole edges versus hole centers). Number of holes in each sample is indicated above the respective bars. Tomography data processed using IMOD (Kremer et al., 1996).

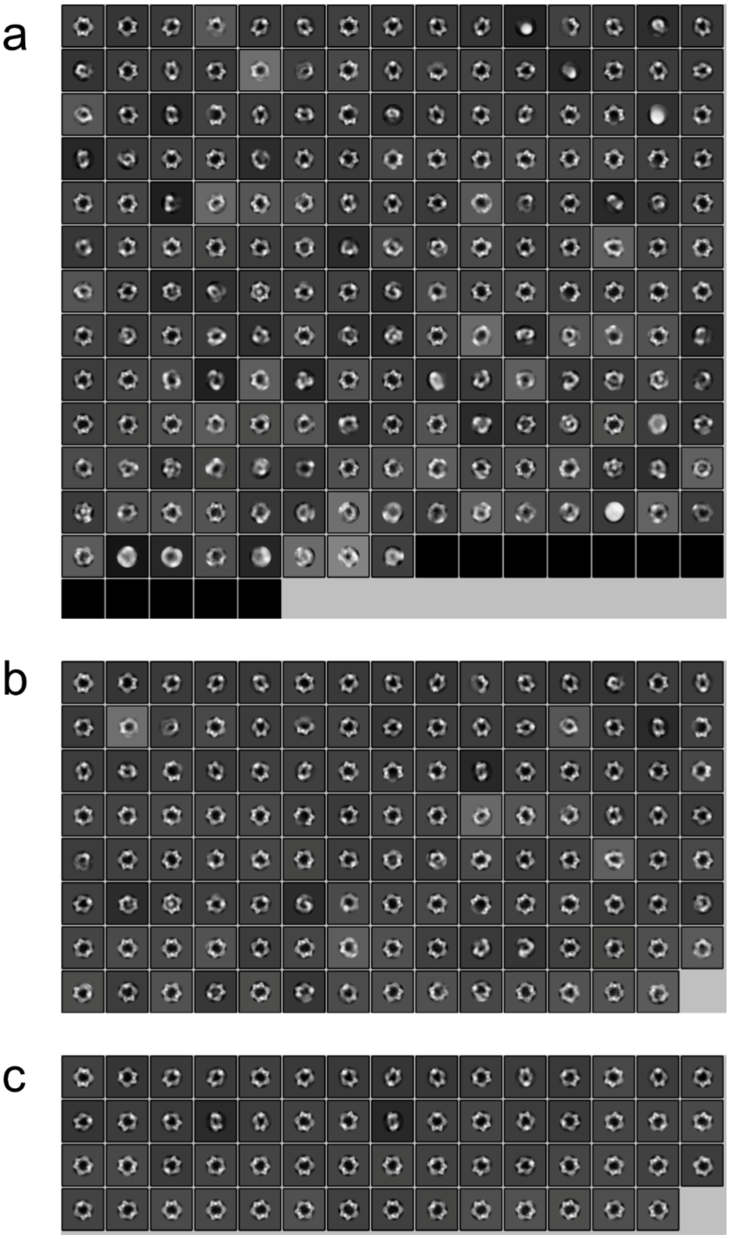

Supplementary Figure 5. Representative example of determination of fraction of intact rings in a data set. (a) All 2D classes (b) All rings (c) Intact rings. The fraction of intact rings was determined by dividing the number of particles contributing to the 2D classes present in (c) by the number of particles contributing to the 2D classes in (b). The data set displayed is from the grid plunged using the chameleon with a 390 ms dispense-to-plunge time. 2D classification was completed using Relion 3.0 (Scheres, 2012).

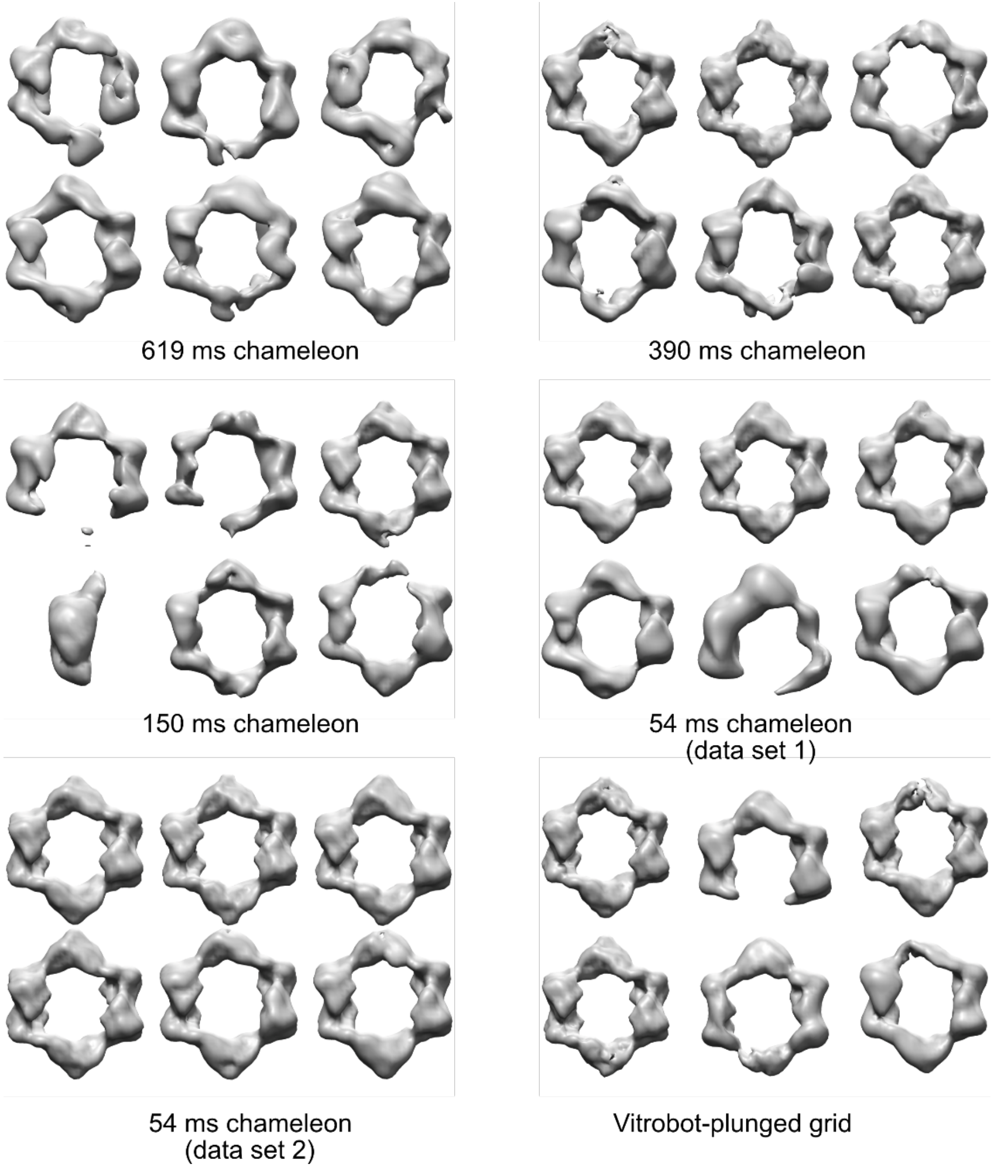

Supplementary Figure 6. 3D classification results for all analyzed data sets. Six classes and 100 rounds of classification were specified in Relion for each data set. Particle inputs to the 3D classification were those that contributed to 2D classes consisting of intact rings or ambiguous side views in each of two rounds of 2D classification. All classes are visualized at an RMSD value of 6. 3D classification was completed using Relion 3.0 (Scheres, 2012).

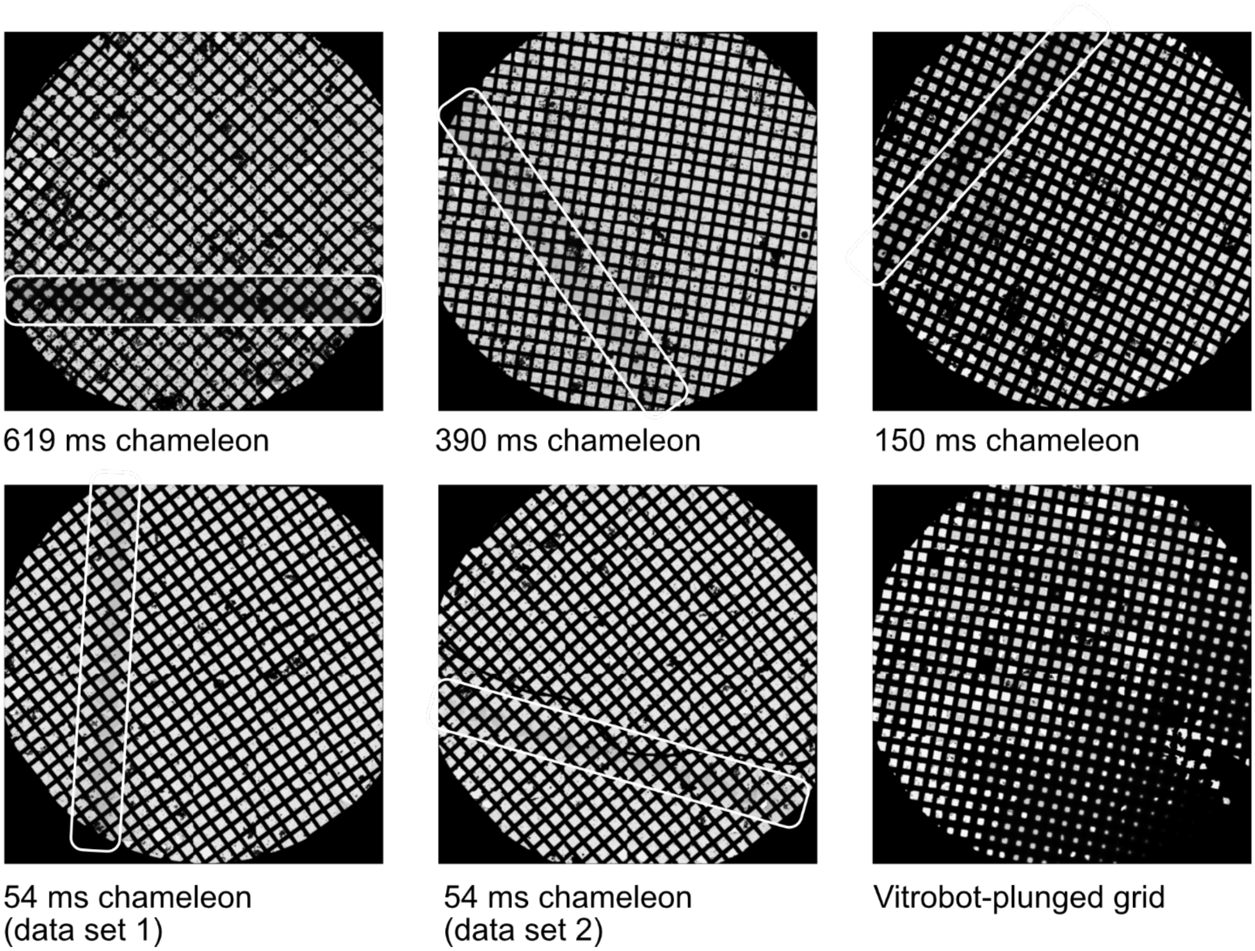

Supplementary Figure 7. Ice thickness varies among the grids used in this study. Atlases taken of each grid that was used to collect a data set. The chameleon-plunged grids have a strip of vitrified sample (indicated with a white rectangle), whereas the Vitrobot-plunged grid has an ice thickness gradient across the grid that is typical of a traditional blotting-based grid preparation technique.

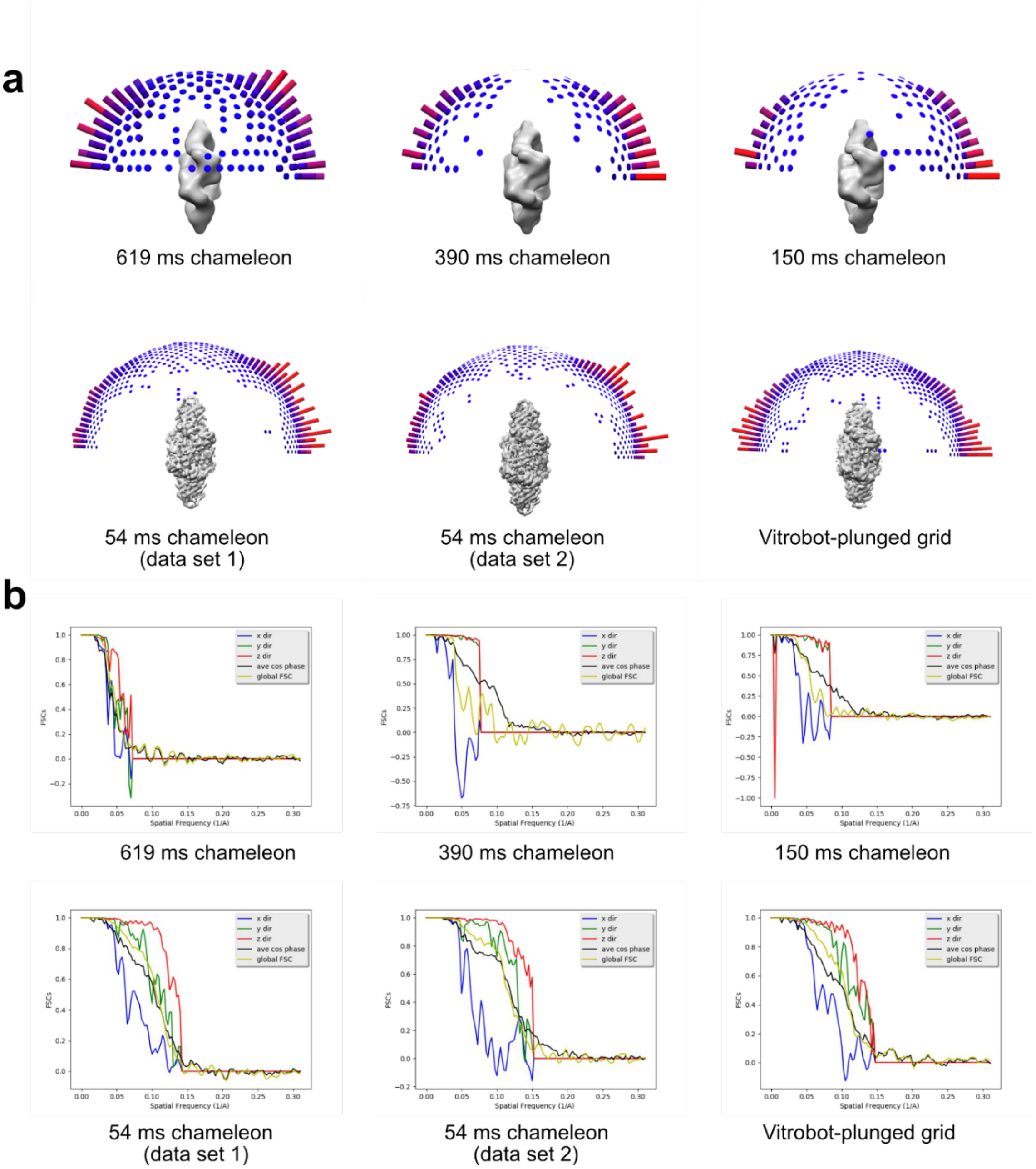

Supplementary Figure 8. Preferred orientation analysis for all data sets analyzed in this study. (a) Angular distribution plots generated in the Relion 3D autorefine program corresponding to data sets from each chameleon grid and the Vitrobot grid visualized in Chimera (Pettersen et al., 2004). Each processed data set contains 7492 particles. The width of the cylinders varies due to the use of different angular sampling parameters for lower resolution versus higher resolution reconstructions. A larger or redder cylinder indicates more particles contributing to that view of the reconstruction. Each map was generated through the Relion postprocess step and is shown at an RMSD value of 6 (Scheres, 2012). (b) 3D FSC plots indicating the Fourier shell correlation as a function of spatial frequency for each data set in the x-direction (blue), y-direction (green), and z-direction (red), as well as the overall global FSC curve (goldenrod) and the average cosine phase (black). Plots were generated using the 3DFSC web server (3dfsc.salk.edu) (Tan et al., 2017).

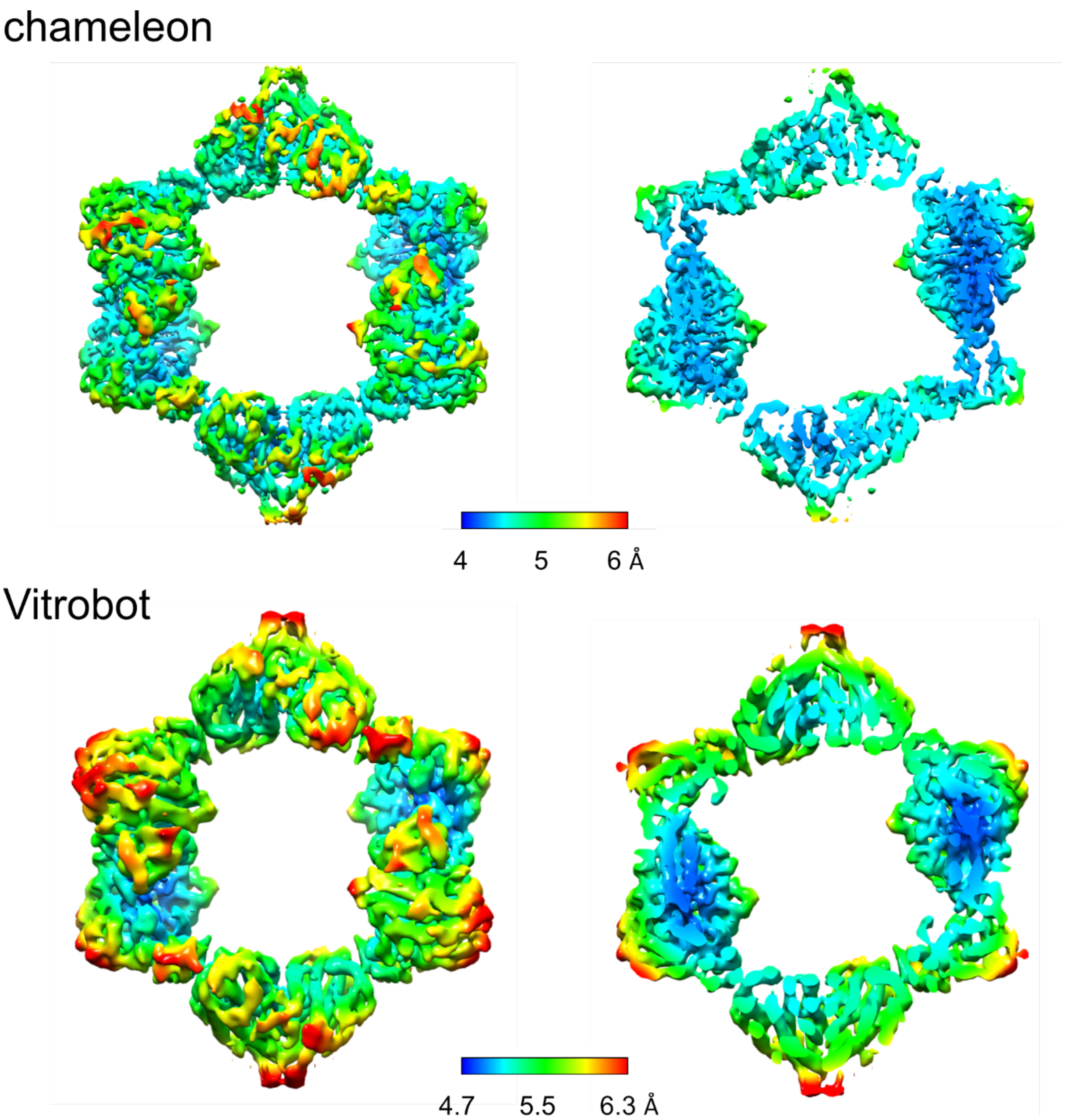

Supplementary Figure 9. Postprocessed maps of the untruncated data sets colored by local resolution. Local resolution map of the data set generated from the chameleon-plunged grid (54 ms dispense-to-plunge time, data set 2; top) and the Vitrobot-plunged grid (bottom). Local resolution was calculated using the Relion LocalRes program and was visualized in Chimera (Pettersen et al., 2004; Scheres, 2012). The two images in each panel on the left and right are a surface view and a central slice of the molecules, respectively.

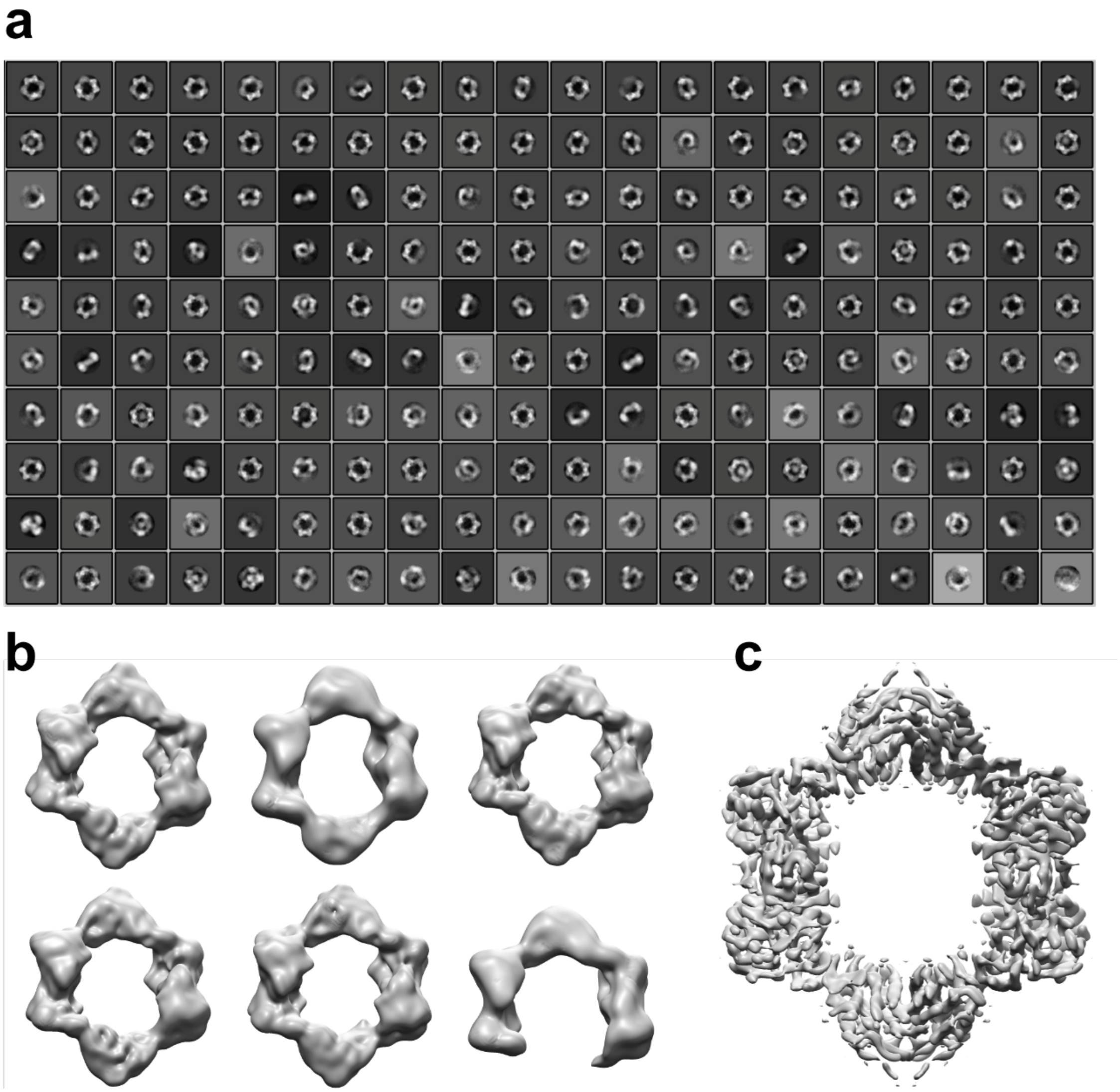

Supplementary Figure 10. A data set collected using a grid plunged on the Vitrobot with a high concentration of glycerol does not process to a high resolution. (a) All 200 2D classes generated from the extra glycerol data set. 2D classes were generated using 25 iterations in Relion 3.0 (Scheres, 2012). (b) 3D classes generated from the extra glycerol data set**.** Six classes and 100 rounds of classification were specified in Relion for classification. Particle inputs to the 3D classification were those that contributed to 2D classes consisting of intact rings or ambiguous side views in each of two rounds of 2D classification. All classes are visualized using Chimera at an RMSD value of 6 (Pettersen et al., 2004). (c) Postprocessed map generated from the data from the extra glycerol Vitrobot-plunged grid that processed to 8.3 Å resolution. Map is visualized at an RMSD value of 6.

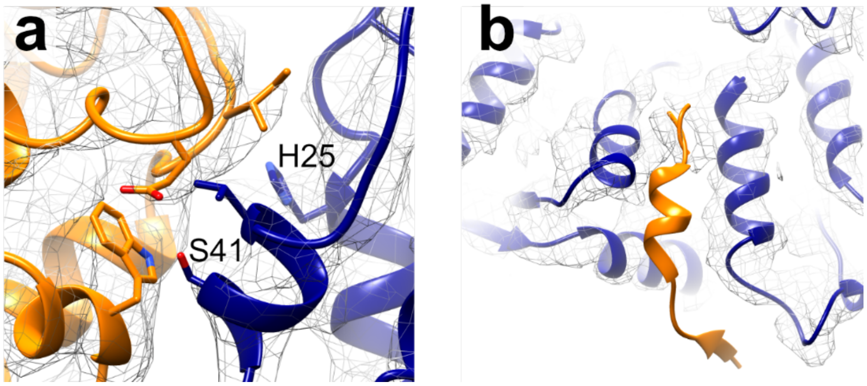

Supplementary Figure 11. Density for α/β interface and β tail residues in the *N. gonorrhoeae* ribonucleotide reductase structure. (a) The density for some residues in the interface region can be seen. Residues H25 and S41, which have been implicated in organism fitness and drug resistance, are labeled. Interface residues are shown as sticks. (b) There is little density present for the *N. gonorrhoeae* β tail residues (short orange loop) despite the tail being structurally visible in *E. coli* crystal structures (long orange helix and sheet). The density in both (a) and (b) is contoured to 6σ. All images generated using Chimera (Pettersen et al., 2004).

Supplementary Table 1. Primers used for amplification of the pET30a(+) backbone in this study.

Primer Name	Sequence (5' -> 3')
prTSL_001	TTGGAAGTAGAGGTTCTCGTGATG
prTSL_002	ACTCGAGCACCACCACCAC

Supplementary Table 2. Gene blocks used for Gibson assembly of the *N. gonorrhoeae* RNR alpha and beta subunits into a pET30a(+) vector. The partial overlap of the hexahistidine tag sequence is underlined, the TEV cleavage tag is indicated in bold, and the protein sequence is not highlighted. DNA after the end of the protein sequence is indicated via lowercase letters. Overlaps with the plasmid backbone or other gene blocks are indicated by grey highlighting.

Gene block name	Identity	Sequence (5′ – 3′)
gbTSL _004	*Ng*RNR alpha, part 1 (codon-optimized)	CATCATCATCAC**GAGAACCTCTACTTCCAA**ATGAACACGCCTACGGATTTAAAGGTAACTAAACGCGATGGGCGTTTAGAAGCTAT<br>CGACTTAGACAAAATCCACCGTGTTGTAACGTGGGCAGCCGAAGGCTTGGAGAATGTCTCTGTATCGCAGGTCGAGCTTAAAAGC<br>CATATCCAATTCTACAACGGGATCCGTACCGATGACATCCACGAAACGATCATCAAGGCGGCGGCCGACCTTATTAGCGAGGATA<br>CGCCCGACTACCAGTACCTGGCCGCACGCCTTGCTATTTTCCACCTTCGCAAAATTGCGTACGGGGAGTACGAGCCTCCCCACCTTT<br>ACAACCACGTCAAGAAACTTACTGATGCAGGAAAGTATGACCGTCACATCCTTGAGGATTATTCACGCGAGGAGTTCGACGAGTT<br>GAACGCTTACATTGACCACGAGCGCGATATGTCGTTTTCGTACGCCGCAGTCAAGCAGTTAGAAGGCAAATATTTGGTCCAGAACC<br>GTGTAACACGTCAAATCTATGAGACTCCTCAATTCCTTTACGTTCTGGTGGCGATGTGTCTTTTTAGCAAGTATCCAAAGGAGGCCC<br>GTTTGGACTATGTCAAGCGTTTCTACGATGCCGTTAGTACATTTAAAGTTTCATTGCCAACCCCGATTATGTCTGGTGTTCGTACCCC<br>TACCCGCCAATTTTCGTCTTGTGTCTTAATCGAGTGCGACGACAGTTTAGATTCAATCAATGCGACCACCTCGGCGATTGTTAAATA<br>CGTGTCGCAACGTGCGGGCATCGGGATTAATGCCGGCCGTATTCGTGGGCTTGATAGTGAAATTCGCGGGGGGGAAGCCCGTCA<br>TACGGGTTGTATTCCATTCTTTAAGATGTTCCAAGCAGCAGTCAAATCTTGCAGTCAGGGCGGTGTTCGCGGGGGGGCTGCGACA<br>TTATTTTATCCATTATGGCACATCGAGGCGGAATCTCTGCTTGTGCTGAAGAACAACCGTGGCGTGGAAGACAACCGTATCCGTCA<br>GCTGGACTACGGAGTTCAGATCAACCGTCTGCTTTACACGCGTTTAATTAAAGGCGGAAACATTACACTTTTTTCGCCAAACGAGG<br>TAAGTGGTCTTTACGAGGCTTTCTTCGCGGACCAGGAtgagttgagcgtcttacaccaaatatga
gbTSL _005	*Ng*RNR alpha, part 2 (codon-optimized)	TGAGTTTGAGCGTCTTTACACCAAATATGAGCAAGATCCTAATATTCGTAAACGCATCATTCCCGCCGCGGACCTGTTTAGTACCTT<br>GATGCAGGAACGCGCGGGTACAGGGCGTATTTACATTCAAAATGTGGACCATTGCAATACTCACTCGCCATTCGATCCTCGTGTAG<br>CGCCTGTCCATCAGTCAAATTTATGCATGGAAATTGCGCTTCCAACCAAACCGCTGGACAACATTAATGATCCCGATGGAGAAATC<br>GCATTGTGTACACTGTCCGCCTTCAACCTGGGTGCACTTAACAGCCTTGATGAGCTGGAGGGATTGGCCGATCTTACTGTACGTGC<br>ATTAGACGCATTGTTGGACTACCAAGGATATCCCGTGGAAGCTGCTCGTACCTCAACAATGGATCGTCGTAGTTTGGGCATCGGTG<br>TTATTAACTACGCATACTACTTAGCTAAAAACGGTGTTCGCTACTCGGATGGATCGGCTTTAGGACTTACCCACCGCACTTTCGAGG<br>CTATTCAATACTATCTGTTGAAGGCTTCAGCCAATTTAGCAAAGGAATACGGCGCTTGTACTTTATTTAATCAGACCGTCTATAGCC<br>AAGGTAAACTGCCCATCGATACTTACAAAAAAGATTTAGATGCGGTATGTGGGGAACCCCTTCATTATGACTGGGAAAGTCTGCG<br>TGCTGATATTGTTAAGTACGGCCTGCGTAATTCCACATTGACCGCTTTGATGCCGAGCGAGACCTCGTCGCAAATCGCGAATGCGA<br>CTAACGGAATTGAACCCCCACGCGGGCTTGTAACGGTGAAGGCGTCGAAAGACGGTATTTTAAAACAGGTGGTCCCGGAGTTCG<br>AGACTCTTAAGAACGCTTATGAGACATTGTGGCAATTACCAGGAAACGAAGGATACCTGAAACTGGTCGGAGTAATGCAAAAATT<br>TGTCGACCAAGCGATTTCGGCGAACACGGCGTATGATCCTGGCAAGTTCGAGGGCAATAAGGTGTCTATGAAACAGATGCTTAAA

		GATTTGTTAACAGCGTACAAATACGGGGTCAAAACGTTGTACTATCACAATACACGTGACGGCGCAGATGACACGCAAACAGACA<br>TCCAAGACGACGGTTGCGCTGGAGGAGCCTGCAAGATCTAAACTCGAGCACCACCACCACCACCACTGAGA
gbTSL_006	*Ng*RNR beta (codon-optimized)	CATCATCATCAC**GAGAACCTCTACTTCCAA**ATGAGCTATAGTACTTTTCCGAAGACCAAGAATGACGCCTTGAAGGAGCCCATGTT<br>TTTCGGGCAGCCAGTAAATGTAGCTCGTTACGACCAGCAAAAGTACGAGGTCTTCGAGAAGCTTATCGAGAAGCAACTGAGCTTC<br>TTTTGGCGTCCCGAAGAAATTGATGTTTCACGCGACCGTATTGACTACGCCAATCTTCCGGAACATGAGAAACATATTTTTATCAGC<br>AATCTTAAGTACCAAACTTTATTGGACTCTATCCAGGGGCGCTCGCCGAATGTAGCACTGCTTCCGTTAGTGAGCATCCCGGAATT<br>GGAAACATGGGTCGAAACTTGGAGTTTTTCTGAGACGATTCATTCTCGCTCTTATACCCACATTATCCGCAATATCGTTAATGACCC<br>TAGTGTTGTTTTCGATGATATCGTAGAGAATGAGTACATCACGGCGCGTGCTGAAGACATCGCTTGCTATTACGACGATCTTATTG<br>AATACACTCAATATTATAATTTATTAGGTGAAGGGGTACATAATGTCGGAGGCAAGCCGGTGACAGTATCCCTTCGTGGACTGAA<br>AAAAAAATTGTATTTATGTCTGATGTGTGTTAACGTCCTTGAAGCAATTCGTTTCTACGTCTCATTTGCCTGTTCCTTTGCCTTCGCT<br>GAACGCGAGTTGATGGAAGGCAATGCAAAAATCATCAAACTGATCGCCCGCGATGAGGCCCTTCATCTGACCGGAACACAGCACA<br>TGTTGAACTTGATGCGCTCAGGTGTCGATGATCCTGAGATGGCGGAAATCGCTGCGGAATTGCAGGACGAATGTTTTCAACTTTTC<br>AAGAAAGCTGCTGAACAAGAGAAAGAATGGGCTGCATATTTGTTTAAAGATGGGTCAATGATCGGCCTTAATAAGGAAATTCTTA<br>GCCAATACGTCGAGTATATTACAAACTTACGCATGCAGGCGGTCGGTCTGCCAGCCGGGTTTGAGGGTGCAAACCAAAACCCTAT<br>TCCGTGGATCAACGCGTGGTTAAGTTCAGATAATGTCCAGGTGGCTCCTCAAGAGGTCGAAATTTCTTCGTACTTGATTGGTCAGA<br>TTGATAGTGAAGTCAATACCGATGACCTGGGCGATTTTGAGCTTTAAactgagcaccaccaccaccactgaga

Supplementary Table 3. Glow discharge times used for each chameleon grid analyzed in this study. All glow discharging was completed at -12 mA.

Dispense-to-plunge time (ms)	Glow discharge time (s)
619	60
390	60
150	140 then an additional 50
54 (data set 1)	350
54 (data set 2)	250

Supplementary Table 4. Data collection parameters for all data sets analyzed in this study.

Microscope / Detector	Talos Arctica G2 / Falcon 3EC
Extraction voltage (V)	4100
Acceleration voltage (kV)	200
Gun lens	5
Spot size (C1)	4
Intensity (C2)	42.255%
Condenser aperture (µm)	50
Objective aperture (µm)	100
Objective lens spherical aberration (Cs) (mm)	2.7
Nominal magnification	92,000
Nominal pixel size (Å)	1.5998
Dose rate on camera (e-/pix/s)	19.37 – 19.42
Exposure time (s)	7.00
Total exposure on specimen (e-/Å2)	53.01 – 53.15
Fractions	14
Defocus values (µm)	-1.2, -1.5, -1.8, -2.1, -2.5, -2.8, -3.1
Shots per hole X Holes per stage shift	1 x 5 (chameleon-plunged grids); 1x1 (Vitrobot-plunged grid)

Supplementary Table 5. Defocus and astigmatism cutoff values for each data set and the resulting micrograph numbers and average drift.

Plunging instrument	Dispense-to-plunge time (ms)	Initial number of micrographs	Defocus cutoff (min – max) (µm)	Astigmatism frequency limit (1/Å)	Drift threshold (min – max) (Å)	Average drift after discarding outliers (Å)	Final number of micrographs
chameleon	619	460	0.997 – 3.33	0.044	1.61 – 14.15	5.28	432
	490	590	0.953 – 3.30	0.088	1.42 – 19.61	5.46	493
	150	365	0.980 – 3.20	0.047	2.11 – 26.07	8.84	321
	54 (data set 1)	320	0.908 – 3.18	0.034	2.08 – 16.13	4.99	290
	54 (data set 2)	620	0.929 – 3.32	0.055	2.00 – 16.91	5.09	580
Vitrobot	n/a	540	0.898 – 3.38	0.058	2.55 – 20.09	6.42	502

Supplementary Table 6. Fraction of initial particles kept at each step of the refinement process for the six data sets analyzed. The cell color of the columns indicates the ranking of the data set as compared to the other data sets at that step, with dark red being the smallest fraction of particles remaining and dark blue indicating the largest fraction of particles remaining.

Plunging Instrument	Dispense-to-plunge Time (ms)	Protein Concentration (mg/mL)	Number of Micrographs in Data Set	Particles Picked (Topaz; 95% Recall Cutoff)	Fraction Particles Remaining After 2D Classification (Round 1)	Fraction Particles Remaining After 2D Classification (Round 2)	Fraction Particles Remaining After 3D Classification
Vitrobot	n/a	0.5	502	104661	0.70	0.66	0.54
chameleon	619	1	432	75444	0.46	0.19	0.13
	390	1	493	132636	0.63	0.56	0.46
	150	1	321	23891	0.52	0.44	0.31
	54	4	290	20460	0.70	0.66	0.63
	54	6	580	55161	0.95	0.91	0.91

Supplementary Table 7. Summary of 3D reconstruction parameters and model refinement of the 54 ms dispense-to-plunge time data set 2. Model refinement was completed using Phenix (Adams et al., 2010).

Particles	
Micrographs used for selection	580
Windowed	55161
In final 3D reconstruction	50425
Symmetry imposed	D2
Map sharpening B-factor	-101.119
Estimated accuracy of rotations (°)	1.286
Estimated accuracy of translations (pix)	0.445
Unmasked resolution at 0.5/0.143 FSC (Å)	4.8 / 4.3
Masked resolution at 0.5/0.143 FSC (Å)	4.4 / 4.2
Local resolution range (Å)	4.2 – 6.2
Number of atoms/residues/molecules	
NCS restrained chains (α; β)	4; 4
Protein atoms, residues per chain (α; β)	5076, 734; 2792, 356
Nucleotide atoms, molecules per chain (α)	87, 3
Mg^{2+} atoms per chain (α; β)	2; 0
Water molecules per chain (α; β)	6; 0
FeO molecules per chain (α; β)	0; 1
Secondary structure restraints (per chain)	
Helices (α; β)	29; 15
Sheets (α; β)	4; 0
Ramachandran (per α/β chain)	1081
Hydrogen bonds (per α/β chain)	267
C-beta torsion restraints (per α/β chain)	2050
Ramachandran angles (%)	
Favored	89.15
Allowed	10.75
Outliers	0.09
r.m.s. deviations	
Bond lengths (Å)	0.004
Bond angles (°)	0.988
Molprobity	
Score	2.15
Clashscore	11.71
Omegalyze outliers	0
B-factors (mix/max/mean)	
Protein	62.33/209.77/108.13
Ligands	98.97/156.94/117.17
Water	98.55/149.57/124.67
EMRinger score	1.14
C-beta outliers (%)	0.00
CaBLAM outliers (%)	5.87
CC (mask)	0.81
Mean CC for ligands	0.83

Supplementary Table 8. Additional sample parameters for chameleon-plunged grids. Relative humidity in the shroud (plunging chamber), shroud temperature, sample volume remaining, and time elapsed since sample aspiration are all recorded by the chameleon during a standard plunging session. The initial aspirated sample volume for all samples was 3 µL.

**Dispense-to-plunge time (ms)**	**Plunge relative humidity (%)**	**Plunge temperature (°C)**	**Sample volume in dispenser (µL)**	**Time elapsed since sample aspiration (mm:ss)**
619	78	23.7	2.85	5 min 05 s
390	51	23.7	2.83	9 min 46 s
150	73	24.6	2.90	13 min 33 s
54 (data set 1)	59	25.6	2.77	6 min 33 s
54 (data set 2)	57	25.6	2.79	1 min 38 s

Supplementary Table 9. Ice thickness calculations for various holes in the Vitrobot-plunged and two fast-plunged chameleon grids as measured by tomography. Ice thickness was measured manually using IMOD by visually identifying the upper and lower faces of the ice and/or particles or contamination on either surface (Kremer et al., 1996).

Plunging instrument	Fast plunge data set	Ice thickness in center of hole (nm)	Ice thickness at edge of hole (nm)
chameleon	1	48.8	83.6
		50.2	68.7
		35.6	56.3
		45.2	62.0
		49.6	64.8
		43.6	67.3
	2	32.3	51.6
		53.2	87.7
		29.2	65.6
		28.4	52.4
		33.6	42.2
		37.8	57.9
		46.3	56.5
		43.9	55.2
		39.4	55.2
		20.1	52.4
		38.6	53.0
Vitrobot	n/a	44.4	60.7
		44.7	56.0
		46.1	50.2
		56.8	48.5
		48.5	49.9
		41.9	49.1
		42.5	51.0
		40.3	53.0
		46.6	40.8
		46.3	49.4
		40.3	37.8
		45.2	49.6
		40.0	48.8
		35.0	37.2

References

Adams, P.D., Afonine, P.V., Bunkóczi, G., Chen, V.B., Davis, I.W., Echols, N., Headd, J.J., Hung, L.-W., Kapral, G.J., Grosse-Kunstleve, R.W., McCoy, A.J., Moriarty, N.W., Oeffner, R., Read, R.J., Richardson, D.C., Richardson, J.S., Terwilliger, T.C., Zwart, P.H., 2010. PHENIX: a comprehensive Python-based system for macromolecular structure solution. Acta Crystallogr D Biol Crystallogr 66, 213–221. https://doi.org/10.1107/S0907444909052925

Bepler, T., Morin, A., Rapp, M., Brasch, J., Shapiro, L., Noble, A.J., Berger, B., 2019. Positive-unlabeled convolutional neural networks for particle picking in cryo-electron micrographs. Nat Methods 16, 1153–1160. https://doi.org/10.1038/s41592-019-0575-8

Kremer, J.R., Mastronarde, D.N., McIntosh, J.R., 1996. Computer Visualization of Three-Dimensional Image Data Using IMOD. Journal of Structural Biology 116, 71–76. https://doi.org/10.1006/jsbi.1996.0013

Moriya, T., Saur, M., Stabrin, M., Merino, F., Voicu, H., Huang, Z., Penczek, P.A., Raunser, S., Gatsogiannis, C., 2017. High-resolution Single Particle Analysis from Electron Cryo-microscopy Images Using SPHIRE. J Vis Exp. https://doi.org/10.3791/55448

Pettersen, E.F., Goddard, T.D., Huang, C.C., Couch, G.S., Greenblatt, D.M., Meng, E.C., Ferrin, T.E., 2004. UCSF Chimera--a visualization system for exploratory research and analysis. J Comput Chem 25, 1605–1612. https://doi.org/10.1002/jcc.20084

Scheres, S.H.W., 2012. RELION: Implementation of a Bayesian approach to cryo-EM structure determination. Journal of Structural Biology 180, 519–530. https://doi.org/10.1016/j.jsb.2012.09.006

Tan, Y.Z., Baldwin, P.R., Davis, J.H., Williamson, J.R., Potter, C.S., Carragher, B., Lyumkis, D., 2017. Addressing preferred specimen orientation in single-particle cryo-EM through tilting. Nat Methods 14, 793–796. https://doi.org/10.1038/nmeth.4347
